# Disentangling direct and indirect genetic pathways to neurodevelopmental risk: brain structure and behavior in a population‐based parent–offspring trio study

**DOI:** 10.1111/jcpp.70184

**Published:** 2026-06-10

**Authors:** Fin J. E. van Uum, Yllza Xerxa, Alexander Neumann, Jean‐Baptiste Pingault, Charlotte A. M. Cecil, Mina Shahisavandi, Koen Bolhuis, Boglarka Kovacs, Henning Tiemeier, Ryan L. Muetzel, Neeltje E. M. van Haren

**Affiliations:** ^1^ Department of Child and Adolescent Psychiatry/Psychology Erasmus University Medical Center Rotterdam The Netherlands; ^2^ Department of Clinical, Educational, and Health Psychology, Division of Psychology & Language Sciences University College London London UK; ^3^ Social, Genetic & Developmental Psychiatry (SGDP) Centre, Institute of Psychiatry, Psychology & Neuroscience King's College London London UK; ^4^ Department of Epidemiology, Erasmus MC University Medical Center Rotterdam Rotterdam The Netherlands; ^5^ Department of Biomedical Data Sciences, Molecular Epidemiology Leiden University Medical Center Leiden The Netherlands; ^6^ Department of Social and Behavioral Sciences Harvard T.H. Chan School of Public Health Boston USA; ^7^ Department of Radiology and Nuclear Medicine Erasmus University Medical Center Rotterdam The Netherlands

**Keywords:** Neurodevelopmental disorders, polygenic scores, intergenerational transmission, brain structure, ADHD, MDD

## Abstract

**Background:**

Neurodevelopmental disorders (NDDs) are highly heritable, yet the pathways linking parental genetic liability to child outcomes remain poorly understood. Traditional designs cannot easily separate genes transmitted to the child from environmental influences shaped by parental genotypes.

**Methods:**

In 3,270 families from the Generation R Study, we applied a mother–father–offspring trio design to disentangle direct genetic effects from indirect parental influences (‘genetic nurture’). Polygenic scores (PGSs) included a transdiagnostic neurodevelopmental score (NDV) and disorder‐specific scores for ADHD, ASD, and major depressive disorder (MDD). Offspring brain structure (cortical surface area, subcortical gray matter, cerebellar volume) at age 9 and behavioral problems at age 14 were assessed. Structural equation modeling was used to test direct, indirect, and brain‐mediated genetic pathways.

**Results:**

Transdiagnostic NDD genetic liability predicted multiple behavioral domains, primarily via direct genetic effects, with similar influence on internalizing and externalizing problems. ADHD PGS effects closely paralleled those of the NDV PGS, whereas ASD and MDD PGSs showed more domain‐specific associations. Notably, maternal, but not paternal, MDD genetic liability was indirectly related to behavioral problems across domains, independent of the child's own genetic risk. This maternal pathway was partly reporter‐specific but robust for attention problems across both parent and child reports. Global brain measures did not mediate any of the identified genetic pathways.

**Conclusions:**

Intergenerational NDD risk is predominantly driven by direct genetic transmission. However, maternal depression liability exerts additional influence through environmentally mediated pathways. The absence of brain‐mediated effects suggests that the gross neural phenotypes examined here do not capture the mechanisms underlying these pathways. Our findings highlight the importance of maternal influences on child NDD outcomes and underscore the need for longitudinal, multimodal research designs that integrate genetic, caregiving, and fine‐grained neural phenotyping data to inform targeted prevention and early intervention strategies for at‐risk families.

## Introduction

Neurodevelopmental disorders (NDDs), including autism spectrum disorder (ASD) and attention deficit hyperactivity disorder (ADHD), are among the most prevalent child psychiatric conditions, affecting educational achievement, employment, health, and social relationships (Scandurra et al., 2019). ASD affects an estimated 0.7–3% of the population, whereas ADHD is more common, with a prevalence of 5–11% (Francés et al., 2022). These conditions frequently co‐occur and share cognitive, social, and emotional features (Morris‐Rosendahl & Crocq, 2020; Thapar, Cooper, & Rutter, 2017). Rather than mapping onto discrete diagnostic boundaries, this overlap is increasingly understood as reflecting shared, pleiotropic genetic liability that spans neurodevelopmental and broader psychopathology domains (Hughes et al., 2023). This perspective motivates integrative frameworks that consider both disorder‐specific and shared genetic and environmental influences.

A central challenge in elucidating NDD etiology is disentangling direct from indirect genetic transmission of risk from parent to child. Direct transmission refers to risk alleles passed from each parent to the child, directly influencing offspring phenotype. Indirect transmission occurs when non‐inherited parental alleles affect child outcomes via environments shaped by parental genetics (Kong et al., 2018). Trio designs, which jointly analyze parent and offspring genomes, allow these effects to be separated (Pingault et al., 2022).

Evidence from genomic parent–offspring trio studies indicates that direct genetic effects explain the majority of variance in neurodevelopmental and related behavioral traits, including ADHD (Cheesman et al., 2020; Eilertsen et al., 2022; Hegemann et al., 2025; Pingault et al., 2022; Voronin et al., 2024). At the same time, these studies also provide evidence for indirect genetic influences operating via the family environment. For example, maternal genetic liability for traits such as neuroticism has been shown to predict child ADHD symptoms beyond inherited risk (Pingault et al., 2022). Clarifying the relative contribution of direct versus indirect pathways is therefore essential, as prevention and intervention strategies may differ depending on whether risk is transmitted primarily through genetic inheritance or through environmentally mediated mechanisms.

The contributions of the present study are twofold. First, we address phenotypic overlap across NDDs by applying a transdiagnostic polygenic score (PGS). PGSs, derived from genome‐wide association studies (GWAS), aggregate common variants weighted by effect sizes (Choi, Mak, & O'Reilly, 2020). Given the strong correlations between PGSs for different NDDs (Li et al., 2020), their genetic architecture reflects both pleiotropy and polygenicity. Accordingly, transdiagnostic scores such as the neurodevelopmental (NDV) PGS, which captures shared risk variance across ADHD, ASD, major depressive disorder (MDD), and Tourette's syndrome (Lee et al., 2019), offer a way to quantify broad NDD liability (Shi et al., 2023). Although MDD is not conventionally classified as an NDD, its genetic risk overlaps with these disorders, loading on the same genetic factor (Lee et al., 2019). To reflect this genetic architecture, we include MDD risk within the NDV PGS, prioritizing genomic patterns over diagnostic categories. By focusing on this transdiagnostic measure, we aim to clarify how shared genetic liability contributes to heterogeneous NDD manifestations during adolescence.

Second, we integrate neurobiological markers into a triadic genetic framework. Structural brain alterations are consistently observed in children and adolescents with NDDs (Hoogman et al., 2019; van Rooij et al., 2017) and tend to correlate with polygenic liability (Alemany et al., 2019). Genes contributing to the NDV factor are preferentially expressed in brain tissue during prenatal development and influence processes such as neurogenesis and synapse formation (Lee et al., 2019). These findings suggest that genetic vulnerability may be expressed through early neurodevelopmental variation that contributes to later‐emerging behavioral profiles, including both NDD‐proximal traits (e.g., attention and social functioning) and broader dimensions of psychopathology. In this context, neuroimaging markers provide an opportunity to examine whether childhood brain structure lies on the developmental pathway linking genetic liability to later behavioral outcomes.

In summary, this study aims to (1) evaluate the transmission profile of a transdiagnostic NDD PGS, (2) assess the relative contributions of direct and indirect genetic pathways, and (3) examine whether childhood brain morphology mediates these associations across adolescence. Mediation analyses are embedded in a developmental framework in which parental and offspring genetic liability precede childhood brain structure and subsequent adolescent behavioral outcomes, enabling assessment of mediation along both direct and indirect genetic pathways. Clarifying these processes may refine our understanding of how genetic and environmental influences intersect in NDD risk, ultimately informing prevention and intervention strategies in child psychiatry.

## Methods

### Sample description

This study draws on data from the Generation R study, a population‐based cohort study located in Rotterdam, the Netherlands (Kooijman et al., 2016). The cohort comprises children born to mothers with expected delivery dates between April 2002 and January 2006, with developmental assessments conducted from fetal life onwards via longitudinal follow‐up. Comprehensive assessments are conducted at each measurement point, including biological samples, neuroimaging, and behavioral evaluations. Genotyping is performed for the child as well as both parents.

From the initial Generation R cohort (*N*_trios_ = 9,901), unique mother–father–offspring triads were identified by randomly excluding siblings from the dataset (*N*_trios_ = 9,153). Only families with complete genetic data, following PGS computation and filtering, were retained, yielding 3,270 unique triads (*N*_participants_ = 9,810). Offspring were aged 12–16 years at the time of questionnaire assessment.

The study received approval from the Erasmus MC Medical Ethical Committee, and informed consent was obtained from both parents and children aged 12 and older. The sample demographics closely reflect the population of Rotterdam, encompassing a wide range of ethnic backgrounds. The primary inclusion criterion for this study was the availability of complete genetic data for all members of the family triad (mother, father, child). Missing data for outcome, mediator, and covariate variables were permitted to maximize the sample size and are addressed in the results section.

### Measures

#### Behavioral assessment

Psychiatric symptoms at age 14 were assessed using the child‐reported *Youth Self‐Report* (YSR) and the parent‐reported *Child Behavior Checklist* (CBCL) (Achenbach & Rescorla, 2001). CBCL questionnaires were completed primarily by mothers (>99%), with the remainder completed by fathers or other caregivers. The YSR contains 112 items and the CBCL 113 items, each rated on a three‐point scale: 0 = *not true*, 1 = *somewhat or sometimes true*, and 2 = *very true or often true*, referring to behaviors over the preceding 2 months. Both instruments adopt a dimensional approach to common mental health problems, such as attention difficulties and social challenges (Krueger et al., 2018).

Our primary focus is on the externalizing and internalizing broadband scales (Achenbach, Ivanova, Rescorla, Turner, & Althoff, 2016). The externalizing scale captures behaviors such as aggression and rule‐breaking, while the internalizing scale assesses symptoms of anxiety and depression. These domains are frequently observed in NDDs and serve as important indicators of symptom severity (Bauminger, Solomon, & Rogers, 2010). In addition, we include the attention problems and social problems syndrome scales, which are particularly relevant for NDD phenotypes: the attention problems subscale is used as a proxy for ADHD symptoms, and the social problems subscale is associated with ASD (Ooi, Rescorla, Ang, Woo, & Fung, 2010). Higher scores indicate greater symptom severity within each domain.

The YSR and CBCL have demonstrated strong psychometric properties, with test–retest reliability coefficients of .92 and .85, and internal consistency values of .94 and .92 (Achenbach, 2010; Achenbach & Rescorla, 2001). Both measures have been validated across 23 societies, including the Netherlands (Ivanova et al., 2010). The use of multiple informants enhances behavioral assessment by enabling cross‐informant comparisons and integrating perspectives from different contexts.

#### MRI image acquisition

The MRI acquisition protocol for Generation R has been described in detail elsewhere (White et al., 2017). In brief, neuroimaging data were collected at age 9 using a 3 Tesla Discovery MR750w scanner (GE Healthcare, Milwaukee, WI, USA) with an 8‐channel receive‐only head coil. A coronal inversion recovery fast spoiled gradient recalled (IR‐FSPGR) sequence with ARC acceleration was used. The key scanning parameters were TR = 8.77 ms, TE = 3.4 ms, TI = 600 ms, flip angle = 10°, field of view (FOV) = 220 × 220 mm, acquisition matrix = 220 × 220, slice thickness = 1 mm, and 230 slices were acquired.

#### MRI image processing

Structural T1‐weighted MRI data were processed using FreeSurfer (v6.0). The following standard preprocessing steps were applied: removal of non‐brain tissue, B1 inhomogeneity correction, whole‐brain tissue segmentation, and cortical surface reconstruction, which underwent visual inspection for quality control (Muetzel et al., 2019). Scans that failed quality control, exhibited significant artifacts, or contained substantial incidental findings were excluded from statistical analysis (*N* = 275).

For each hemisphere, global brain metrics were extracted and averaged across hemispheres to yield single values for cerebellum volume, subcortical gray matter volume, and cortical surface area. These metrics were selected a priori based on both empirical and mechanistic considerations. Empirically, neuroimaging studies of neurodevelopmental disorders tend to report small but widely distributed structural differences at the global level (Hoogman et al., 2019; Van Rooij et al., 2017). Mechanistically, gene‐expression analyses indicate that genetic variants contributing to the NDV cluster show preferential expression in the cerebellum, particularly during early brain development (Hughes et al., 2023).

#### Genotype data preprocessing and quality control

Genotype data for the Generation R Study were generated using Illumina 610 K and 660 K arrays for GenR3 children, GSA‐MD v2.0 arrays for GenR4 children, and GSA‐MD v2.0/v3.0 arrays for parents (Ghatan et al., 2024; Jaddoe et al., 2007; Medina‐Gomez et al., 2015). Standard quality control (QC) procedures were applied, including filters for call rate, Hardy–Weinberg equilibrium, excess heterozygosity, and concordance between genetically inferred and reported sex and relatedness.

Genotype imputation was conducted using the 1,000 Genomes Project reference panel (Phase 3, Version 5) (Delaneau, Marchini, & Zagury, 2011; Howie, Donnelly, & Marchini, 2009). Post‐imputation, autosomal SNPs were retained if they had an imputation quality score (R^2^) ≥ 0.80 and minor allele frequency (MAF) ≥ 1%. This resulted in 7,673,245 SNPs for participants genotyped on Illumina 610 K/660 K arrays (GenR3), 9,508,795 SNPs for GSA‐MD v2.0 (GenR4), and 9,520,497 SNPs for the parent cohort.

#### PGS calculation and harmonization

Polygenic scores were calculated using LDpred2‐auto (Privé, Arbel, & Vilhjálmsson, 2020), based on the largest available GWAS summary statistics of European ancestry for major phenotypes: major depressive disorder (MDD) (Adams et al., 2024), ADHD (Demontis et al., 2023), and ASD (Grove et al., 2019). Due to limited GWAS sample size, an individual disorder‐specific PGS was not computed for Tourette syndrome.

The NDV PGS was based on previously published work by Hughes et al. (2023). In that study, genomic structural equation modeling (gSEM) was used to model the genetic covariance structure across multiple psychiatric disorders, identifying a latent genetic factor capturing shared liability across ADHD, ASD, MDD, and Tourette syndrome. For this latent factor, gSEM yields SNP‐level effect estimates representing each variant's contribution to NDV, derived as linear combinations of disorder‐specific GWAS effects weighted by their respective factor loadings.

Imputed genotype dosage data in PLINK BED format served as input for the computation of individual‐level PGSs. Linkage disequilibrium (LD) was modeled using the European‐ancestry UK Biobank LD reference panel (1,054,330 HapMap SNPs), as recommended by LDpred2 developers (Privé, Albiñana, Arbel, Pasaniuc, & Vilhjálmsson, 2023).

Across the GWAS summary statistics and LD reference panel, approximately 924,000 to 979,000 SNPs overlapped with those available in GenR3 children, 964,000 to 1,036,000 SNPs in GenR4 children, and 964,000 to 1,036,000 SNPs in the parent cohort, ensuring consistent LD estimation and alignment with GWAS ancestry across all subcohorts.

#### Ancestry adjustment and harmonization

Given the multi‐ethnic composition of the cohort, 20 genomic components were derived to capture population structure, via multidimensional scaling (MDS) for children and principal component analysis (PCA) for parents using PLINK software (Chang et al., 2015; Purcell et al., 2007).

To correct for residual ancestry differences and potential batch effects, PGS values were residualized and z‐standardized within each genotyping subcohort (GenR3 children, GenR4 children, and GenR parents), using the first 20 genomic components. Residualization accounts for ancestry‐ and platform‐specific variation, while z‐standardization controls for differences due to minor variations in SNP availability or genotyping batch. These steps minimize confounding from population stratification and technical artifacts. Scripts for genotype preprocessing and PGS computation are publicly available (GitHub link).

### Statistical analysis

We examined relationships among genetic, neuroimaging, and behavioral data using structural equation modeling (SEM) implemented in the *lavaan* package (version 0.6.17) (Rosseel, 2012) in R (version 4.3.2). Descriptive statistics (*N*, mean, SD, minimum, maximum, median) were calculated for quantitative variables; categorical demographics are reported as frequencies (*N*, %). All variables were standardized prior to model estimation.

SEM models were estimated using maximum likelihood with robust standard errors (MLR) to accommodate non‐normality in behavioral data. Missing data were handled using a two‐tiered approach: listwise deletion was applied for descriptive statistics and correlation analyses, whereas all SEMs were estimated using full‐information maximum likelihood (FIML), allowing all available data to contribute to parameter estimation.

Within the FIML framework, auxiliary demographic variables (mother's education, partner's education, paternal age at conception, and marital status) were included only to improve estimation of missing outcome and neuroimaging data under a missing‐at‐random assumption. These variables were not included as covariates in the structural models and therefore did not condition the estimated genetic pathways.

In the genetic models, covariates were restricted to the child's age at assessment and maternal age at conception. Other demographic characteristics, including parental socioeconomic indicators, were not included as covariates because they are partially influenced by parental genotype and may therefore lie on the indirect genetic pathway rather than function as exogenous confounders in a trio design. Conditioning on such variables would risk attenuating indirect genetic effects. Intracranial volume (ICV) was included in all models involving brain metrics. Behavioral outcomes were residualized for child sex prior to modeling.

The analytic approach began with models testing simple genetic–behavioral and genetic–MRI associations (Figure 1a). These were followed by genetic trio models that simultaneously estimated direct genetic effects (child PGS → outcome) and indirect effects (parental PGS → outcome) (Figure 1b). Finally, trio mediation models incorporated paths from child and parental PGSs to brain metrics and from brain metrics to behavioral outcomes, with indirect effects calculated as the product of PGS → MRI and MRI → outcome paths for each genetic pathway (Figure 1c). In this way, the approach separates direct and indirect genetic influences into brain‐mediated and non–brain‐mediated effects. Mediation effects are reported, with total effects corresponding to those found in the trio models.

**Figure 1:**
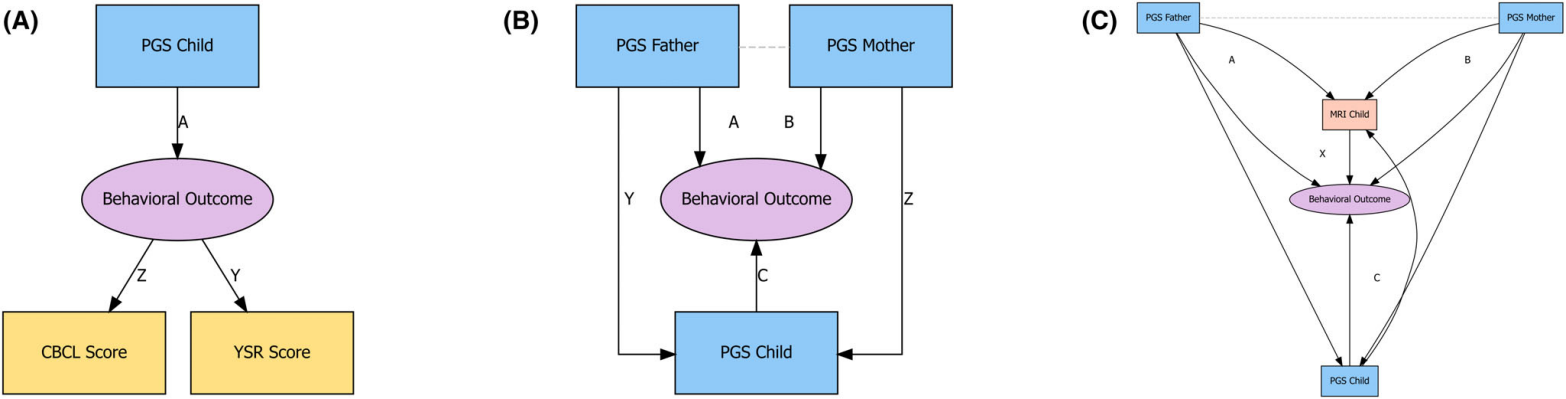
Schematic representation of the SEM models. Panel A: Initial association models testing total associations between child polygenic scores (PGSs; NDV, ADHD, ASD, or MDD, modeled in separate models) and adolescent outcomes. Behavioral outcomes were operationalized at the domain level (internalizing, externalizing, attention problems, social problems) and modeled as a latent factor capturing shared variance between parent‐reported CBCL and self‐reported YSR domain scores. The YSR loading (path Y) was fixed to 1, and the CBCL loading (path Z) was freely estimated. Path A denotes the association between the child PGS and the latent behavioral outcome. Analogous association models were also estimated for neuroimaging outcomes, in which each brain metric (cortical surface area, subcortical gray matter volume, or cerebellar cortical volume) was modeled as a single observed outcome regressed on the child PGS. Panel B: Genetic trio model illustrating direct genetic effects (path C: child PGS → behavioral outcome) and indirect genetic effects (paths A and B: parental PGSs → behavioral outcome). Genetic inheritance paths (Y and Z) are centered around 0.5. Assortative mating is modeled via covariance between maternal and paternal PGSs (dotted gray line). Behavioral outcomes follow the latent factor structure described in Panel A. Panel C: Genetic trio mediation model extending Panel B by introducing a neuroimaging measure as a mediator of genetic effects on behavior. Separate paths are specified from the father's, mother's, and child's PGSs to the neuroimaging variable (paths A, B, and C, respectively), and from the neuroimaging variable to the behavioral outcome (path X). Mediation effects are tested separately for each genetic pathway and are defined as the products AX (paternal), BX (maternal), and CX (child). Behavioral outcomes follow the same latent‐factor specification as in Panels A and B. CBCL, Child Behavior Checklist; PGS, Polygenic Score; YSR, Youth Self‐Report

In total, four primary regression models, four trio models (one per behavioral outcome), and 12 mediation models (four behavioral outcomes × three brain metrics) were estimated. In all primary analyses, behavioral problems were modeled as latent factors capturing shared variance between CBCL and YSR domain scores, with the YSR loading fixed at 1 and the CBCL loading freely estimated to allow for scaling differences between informants.

Single‐informant outcomes (CBCL‐only or YSR‐only) were used exclusively in predefined sensitivity and robustness analyses, which also examined individual PGS components (ADHD, ASD, MDD). In an additional sensitivity analysis, paternal age was included as a covariate alongside maternal age in the models. Maternal and paternal age at conception were moderately correlated within couples (*r* = 0.63; *N* = 3,265), leaving a substantial proportion of variance in paternal age independent of maternal age. Statistical significance was defined as *p* < .05.

False discovery rate (FDR) correction was applied a priori within clearly defined families of hypothesis tests, reflecting both the polygenic score (transdiagnostic vs. disorder‐specific) and the genetic pathway under investigation (direct child, maternal indirect, paternal indirect effects). Specifically, FDR correction was performed separately for (i) primary association models, (ii) trio transmission models, and (iii) trio mediation models, and within trio models was applied independently to direct and indirect genetic pathways rather than across all parameters jointly. This strategy was chosen to control type I error while preserving power to detect indirect genetic effects. Effect sizes are reported as standardized regression coefficients. Full model specifications are provided in Appendix S1, with a summary of model classes, covariates, and FDR families provided in Table S1. Complete results (i.e., uncorrected and unstandardized estimates) are reported in Appendix S2.

## Results

### Sample characteristics

Among the 3,270 children, behavioral data were available for 1,993 participants based on parent reports (CBCL) and for 1,904 participants based on child self‐reports (YSR). Usable neuroimaging data were obtained from 1,413 children. Demographic characteristics and descriptive statistics are presented in Table 1.

**Table 1:** Sample characteristics and descriptive statistics of the study population

Variable	*N*	Mean (SD)/ %	Range	Median	Missing as %
Sample characteristics					
Age of mother at conception	3,270	30.25 (4.75)	16–44	30.70	0
Age of father at conception	3,265	32.87 (6.30)	17–58	32.78	0.15
Age of child at clinical assessment	1970	13.61 (0.42)	12–16	13.51	39.76
Sex child					
Boy	1,639	50.12	–	–	–
Girl	1,631	49.88	–	–	–
Behavioral outcomes					
Social problems (YSR)	1903	3.12 (2.34)	0–14	3	41.80
Social problems (CBCL)	1990	1.28 (1.85)	0–12	1	39.14
Attention problems (YSR)	1904	4.97 (3.19)	0–16	5	41.77
Attention problems (CBCL)	1990	3.30 (3.14)	0–17	3	39.14
Internalizing problems (YSR)	1903	8.82 (7.08)	0–40	7	41.80
Internalizing problems (CBCL)	1993	5.46 (5.76)	0–41	4	39.05
Externalizing problems (YSR)	1901	6.84 (5.14)	0–31	6	41.87
Externalizing problems (CBCL)	1986	4.07 (4.95)	0–46	2	39.27
Neuroimaging variables					
Cerebellar cortical volume (mm^3^)	1,413	59,388 (5060)	37,631–79,199	59,339	56.79
Subcortical gray matter volume (mm^3^)	1,413	60,762 (4516)	45,932–76,673	60,661	56.79
Cortical surface area (mm^2^)	1,413	93,832 (8884)	64,933–120,734	93,558	56.79
Estimated intracranial volume (mm^3^)	1,413	1,521,685 (139072)	1,060,800–1,922,080	1,521,020	56.79
Marital status parents					
Married/Living together	2,889	88.35	–	–	–
No partner	230	7.03	–	–	–
NA	151	4.61	–	–	–
Education mother					
No education finished	4	0.12	–	–	–
Primary	221	6.76	–	–	–
Secondary, phase 1	382	11.68	–	–	–
Secondary, phase 2	917	28.04	–	–	–
Higher, phase 1	718	21.96	–	–	–
Higher, phase 2	875	26.76	–	–	–
NA	153	4.68	–	–	–
Education father					
No education finished	0	0	–	–	–
Primary	213	6.51	–	–	–
Secondary, phase 1	375	11.47	–	–	–
Secondary, phase 2	735	22.48	–	–	–
Higher, phase 1	566	17.31	–	–	–
Higher, phase 2	904	27.65	–	–	–
NA	477	14.59	–	–	–

### Preliminary analyses

Preliminary analyses showed moderate correlations between parent‐reported (CBCL) and self‐reported (YSR) behavioral outcomes (*r* = .41–.55), as well as moderate correlations across behavioral domains (*r* = .23–.64; Figure 2a; Table S2). Genetic validity checks revealed the expected correlations between parental and offspring polygenic scores (*r* = .48–.51), confirming valid intergenerational genetic links (Figure 2b; Table S3). Correlations among disorder‐specific PGSs were generally modest (e.g., ADHD–ASD: *r* = .29; ADHD–MDD: *r* = .27; ASD–MDD: *r* = .13), whereas the transdiagnostic NDV PGS showed stronger associations with its constituent PGSs (ADHD: *r* = .66; ASD: *r* = .54; MDD: *r* = .44; Figure 2b; Table S3).

**Figure 2:**
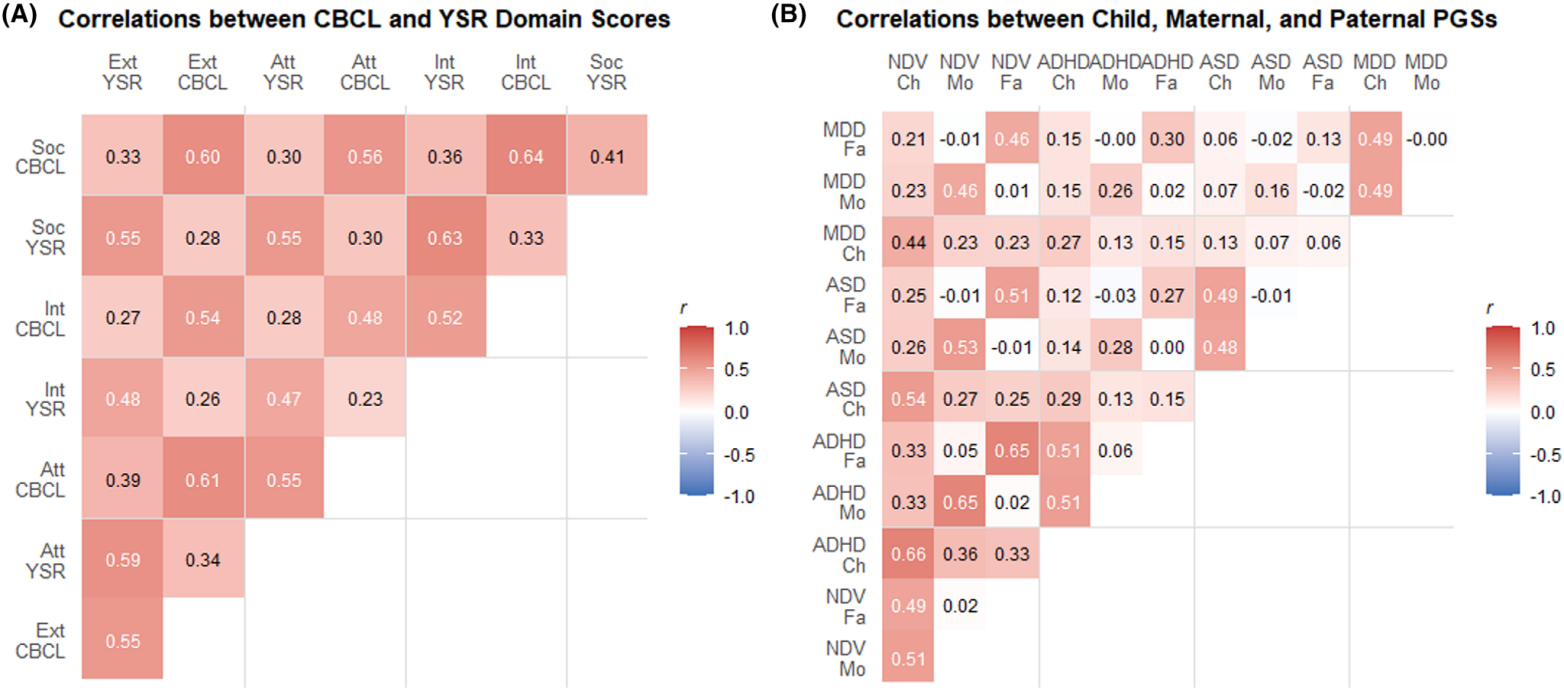
Correlation matrices of behavioral outcomes and polygenic scores. Panel A: Pairwise correlations between parent‐reported (CBCL) and self‐reported (YSR) behavioral domain scores (internalizing (Int), externalizing (Ext), attention (Att), and social (Soc) problems). Panel B: Pairwise correlations among all computed PGSs across children (Ch), mothers (Mo), and fathers (Fa), including the neurodevelopmental (NDV) PGS and disorder‐specific PGSs (ADHD, ASD, MDD). CBCL, Child Behavior Checklist; PGS, Polygenic Score; YSR, Youth Self‐Report

### Association models

Initial association models (Figure 1a) indicated significant relationships between the child NDV PGS and all behavioral domains. The strongest effect was observed for externalizing problems (β = .178, SE = .027, *p* = 1.67 × 10^−12^), followed by attention problems (β = 0.159, SE = 0.026, *p* = 2.87 × 10^−7^), internalizing problems (β = .149, SE = .029, *p* = 5.75 × 10^−7^), and social problems (β = .122, SE = .031, *p* = .008). All associations remained significant after FDR correction for four comparisons (Tables S4a and S4b). Disorder‐specific PGSs were also significantly associated with behavioral outcomes after FDR correction for 12 comparisons (3 PGSs × 4 outcomes), except for the ASD PGS and attention problems (β = .026, SE = .029, *p*_FDR = .321; Tables S5a and S5b).

The child NDV PGS showed no significant associations with brain structure metrics, including total cortical surface area (β = −0.031, SE = 0.014, *p*_FDR = 0.078), cerebellar volume (β = −.022, SE = .022, *p*_FDR = .462), or subcortical gray matter volume (GMV) (β = −.003, SE = 0.017, *p*_FDR = .875). Among disorder‐specific PGSs, only the MDD PGS was significantly associated with brain structure, showing a negative association with subcortical GMV (β = −.051, SE = 0.017, *p*_FDR = .018) after correction for nine comparisons (3 PGSs × 3 brain metrics; Tables S6a and S6b).

### Main analysis 1: Direct and indirect genetic transmission

Using trio models, child, father, and mother PGSs were simultaneously related to child behavioral outcomes, applying a latent factor structure consistent with the initial association models (Figure 1b). Significant direct effects of the child's NDV PGS on behavioral problems were observed: externalizing (β = .140, SE = .037, *p*_FDR = .001), attention (β = .128, SE = .038, *p*_FDR = .006), social (β = .116, SE = .041, *p*_FDR = .024), and internalizing problems (β = .100, SE = 0.038, *p*_FDR = .015) (Figure 3; Tables S7a and S7b). No significant indirect effects were detected for maternal or paternal NDV PGSs, and parental NDV PGSs were uncorrelated (β = .022, SE = .017, *p*_FDR = .202).

**Figure 3:**
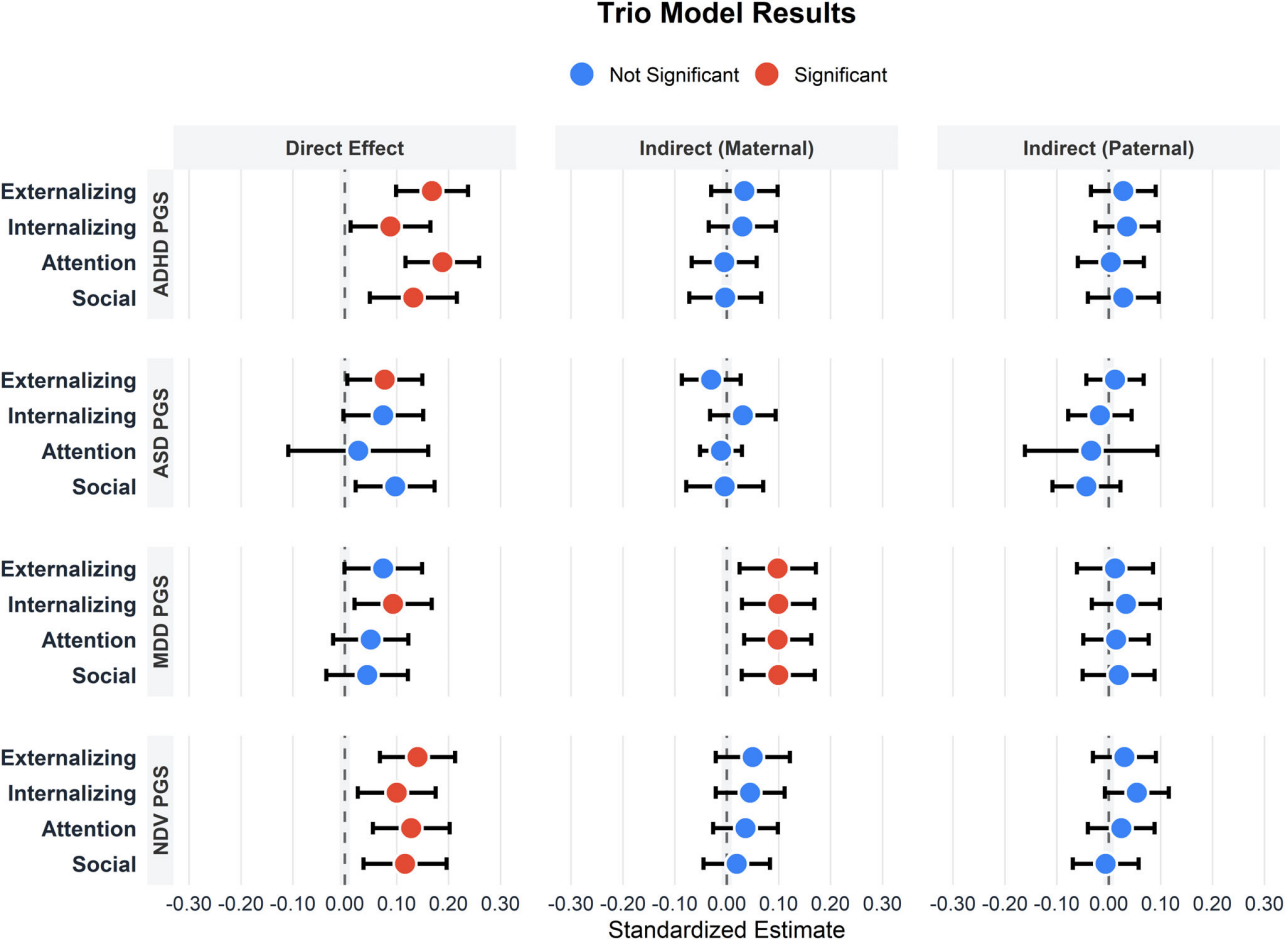
Genetic Trio model results. Standardized effect size estimates are shown for each PGS across behavioral domains (social, attention, internalizing, externalizing) and transmission types (direct, indirect maternal, indirect paternal). Points represent estimated effects, and horizontal bars denote 95% confidence intervals (±1.96 SE). The dashed vertical line indicates zero. Color encodes statistical significance (*p*_FDR < .05; red = significant, blue = not significant). Direct effects were observed for several PGSs, whereas significant indirect effects were limited to the maternal MDD PGS across domains. NDV, Neurodevelopmental Cluster; PGS, Polygenic Score

Sensitivity analyses of disorder‐specific PGSs revealed a similar pattern for the ADHD PGS, with significant direct effects on attention (β = .188, SE = .036, *p*_FDR = .00006), externalizing (β = .168, SE = .035, *p*_FDR = .00006), social (β = .132, SE = .043, *p*_FDR = .012), and internalizing problems (β = .088, SE = .039, *p*_FDR = .038), but no indirect effects. The ASD PGS showed direct effects only on externalizing problems (β = .077, SE = .037, *p*_FDR = .038), with no significant effects on other domains or indirect effects. The MDD PGS exhibited direct effects only on internalizing problems (β = .093, SE = .038, *p*_FDR = .038). Notably, significant maternal indirect effects of the MDD PGS were observed across all behavioral domains; social (β = .099, SE = .036, *p*_FDR = .036), attention (β = .098, SE = .033, *p*_FDR = .018), internalizing (β = .099, SE = .036, *p*_FDR = .018), and externalizing problems (β = .098, SE = .037, *p*_FDR = .020), independent of the child PGS. No paternal indirect effects were detected. All *p*‐values were corrected for 12 comparisons (4 outcomes × 3 PGSs; Tables S8a and S8b).

Follow‐up analyses separated parent‐ and self‐reported outcomes to assess potential rater bias in maternal MDD PGS indirect effects (Figure 4; Tables S9a and S9b). Most maternal indirect effects were specific to parent reports; no significant indirect effects were observed for self‐reported externalizing, internalizing, or social problems (*p*_FDR > .05). However, maternal indirect effects on attention problems were present in both parent‐ and self‐reports. These analyses included eight comparisons (2 raters × 4 outcomes), with FDR correction applied.

**Figure 4:**
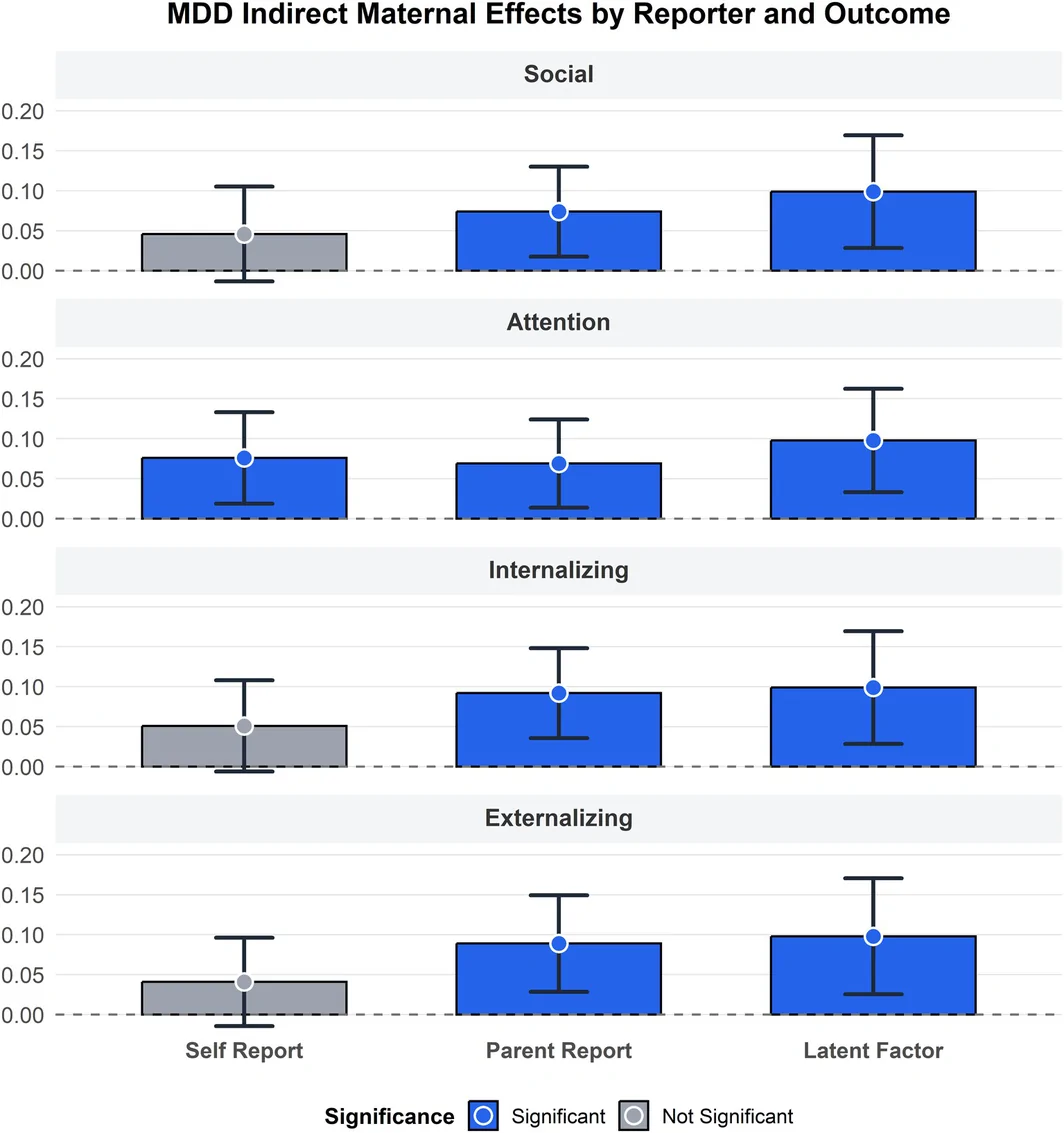
Maternal indirect genetic effects of the MDD PGS across raters. Standardized effect size estimates for maternal indirect effects of the MDD PGS on behavioral domains are shown by reporter: latent factor (combined CBCL and YSR), self‐report (YSR), and parent report (CBCL). Error bars denote 95% confidence intervals (±1.96 SE). Significance was assessed with FDR‐adjusted *p*‐values (blue = significant; gray = not significant). All latent‐factor effects were significant. Attention showed significant effects in both self‐ and parent reports, whereas social, internalizing, and externalizing were significant only in parent reports. CBCL, Child Behavior Checklist; MDD, major depressive disorder; PGS, polygenic score; YSR, Youth Self‐Report

### Main analysis 2: Neural mediation effects

Trio mediation models (Figure 1c) revealed no significant mediation effects of brain structure on the relationship between PGSs and behavioral outcomes, indicating that structural brain measures did not account for the genetic effects observed in the trio models (Tables S10a and S10b). In exploratory analyses separating parent‐ and self‐reports (24 comparisons: 2 raters × 3 brain metrics × 4 outcomes), modest mediation effects appeared but did not survive FDR correction. For example, the direct genetic effect of the NDV PGS on parent‐reported internalizing problems showed suggestive mediation by cerebellar volume (uncorrected *p* = .046; β = .011, SE = .006), which was non‐significant after correction (*p*_FDR = .57) (Figure 5).

**Figure 5:**
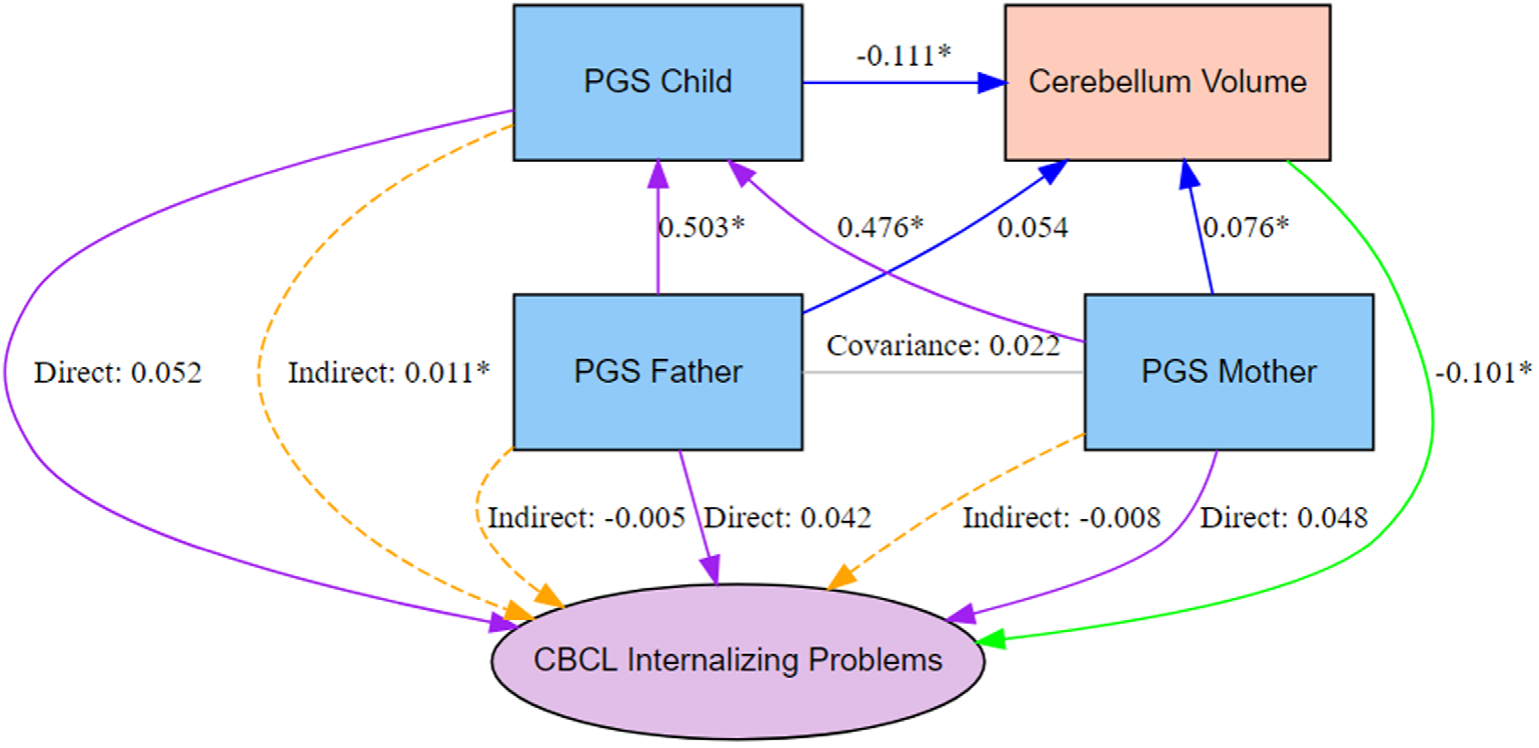
Trio Mediation Model: NDV PGS on Internalizing Problems through Cerebellar Volume. Schematic of the mediation model linking the child's NDV PGS to parent‐reported internalizing problems, with cerebellar volume as a mediator. Both direct and indirect effects across child, mother, and father transmission pathways are modeled, accounting for genetic inheritance and assortative mating. Mediation via cerebellar volume is indicated by dashed yellow lines. The direct genetic effect (std. β = .063) decomposes into a direct effect independent of cerebellar volume (std. β = .052) and an indirect effect via cerebellar volume (std. β = .011). Specifically, the child's NDV PGS negatively associates with cerebellar volume (std. β = −.111), which in turn negatively relates to internalizing problems (std. β = −.101). This mediation effect did not survive FDR correction. Significance before correction is indicated by an asterisk (*). CBCL, Child Behavior Checklist; NDV PGS, Neurodevelopmental Cluster; PGS, Polygenic Score

### Sensitivity analysis: Paternal age

Incorporating paternal age at conception as a covariate, alongside maternal age, did not significantly alter the overall results, and all inferences remained consistent with the previous models. For further details, please refer to Appendix S2.

## Discussion

This study employed a genetic trio design to address three central questions: (1) how transdiagnostic genetic risk for neurodevelopmental disorders (NDDs) relates to adolescent behavioral problems, (2) the relative contributions of direct versus indirect genetic transmission, and (3) whether childhood brain structure mediates these effects.

Across analyses, genetic associations with adolescent behavior were driven predominantly by direct effects of the child's genotype. This pattern is consistent with evidence from genomic parent–offspring trio studies indicating that common genetic variation underlying neurodevelopmental traits is transmitted primarily through direct inheritance, while indirect parental genetic effects tend to be more trait‐specific rather than widespread (Cheesman et al., 2020; Eilertsen et al., 2022; Hegemann et al., 2025). Within this transmission framework, the broad phenotypic reach of the transdiagnostic NDV PGS is consistent with genome‐wide evidence for pleiotropy and shared genetic architecture across neurodevelopmental conditions, whereby directly inherited genetic liability contributes to multiple correlated symptom domains (Demontis et al., 2023; Grove et al., 2019).

Disorder‐specific PGSs largely followed this general pattern but also revealed meaningful differentiation: ADHD showed the strongest and most consistent direct associations across behavioral domains, whereas ASD‐related genetic risk was confined to externalizing problems and MDD genetic risk was selectively associated with internalizing symptoms. Notably, the MDD PGS was the only score to exhibit evidence of indirect transmission, with maternal (but not paternal) genetic liability predicting child behavioral outcomes across domains, highlighting that indirect genetic effects may emerge in specific phenotypic contexts rather than as a general feature of neurodevelopmental risk.

### Maternal MDD risk and child development

By distinguishing direct from indirect transmission, our study points to a maternal‐specific pathway through which genetic liability for depression contributes to offspring difficulties. These indirect effects suggest that part of the covariance between maternal risk and child behavior does not reflect shared inherited alleles, but rather environmental conditions shaped by maternal genetic predispositions. Maternal depression risk may influence parenting behaviors, stress regulation, or attachment processes (Bernard‐Bonnin, 2004). Broader contextual factors, such as marital instability and socioeconomic disadvantage, may amplify these influences (Fergusson & Lynskey, 1993). In this sense, maternal genetic liability for MDD may function not only as a biological risk but also as a proxy for intergenerational social determinants of mental health, shaping the environments in which children grow up and broadly contributing to attention, internalizing, and externalizing difficulties.

The absence of paternal indirect effects strengthens the case for maternal specificity. Mechanisms may operate both prenatally, via stress hormones, medication, or substance use during pregnancy (Davis et al., 2007; Koc, Marroun, Stricker, Muetzel, & Tiemeier, 2023), and postnatally, through the quality of mother–child interactions, consistent with mothers' typically greater caregiving involvement in contemporary families (Connell & Goodman, 2002; Pietikäinen et al., 2019). These results highlight maternal risk pathways as potential leverage points for early intervention, particularly in families where maternal depression risk is elevated.

An important caveat is potential reporter bias. Maternal depression risk may influence how mothers perceive and report their children's behavior, potentially inflating problem scores (Berg‐Nielsen, Vika, & Dahl, 2003; Gartstein, Bridgett, Dishion, & Kaufman, 2009). In the present sample, the vast majority of CBCL assessments were completed by mothers, resulting in a highly unbalanced informant distribution. As a consequence, stratifying analyses by parent informant was not feasible, as father‐completed CBCLs were too sparse to yield stable or interpretable estimates.

Consistent with the possibility of reporter bias, most maternal indirect effects were attenuated when child self‐reports were used instead of parent reports. In contrast, attention problems remained significantly associated across both informants, suggesting that this domain may be less susceptible to rater‐specific bias and may reflect a more robust intergenerational association. Taken together, these findings indicate that the observed maternal indirect effects are robust within the primary modeling framework but should be interpreted cautiously, as they may reflect a combination of environmentally mediated genetic influences, informant‐specific processes, and limited statistical power rather than a single explanatory mechanism.

### Brain structure and genetic transmission

We found no evidence that global brain metrics mediated genetic effects on adolescent behavior. This finding should be interpreted in light of the fact that neurodevelopmental disorder–related neuroimaging differences are typically subtle and broadly distributed, particularly in population‐based samples. Although structural deviations in cortical surface area, subcortical regions, and the cerebellum have been reported in NDDs (Hoogman et al., 2019; Van Rooij et al., 2017), the weak associations observed here are consistent with prior work showing that univariate global volume measures often show limited correspondence with behavioral outcomes and may fail to capture distributed or microstructural substrates of genetic liability (Genon, Eickhoff, & Kharabian, 2022). More sensitive neural phenotypes, such as multimodal imaging approaches (Vieira et al., 2024), normative modeling of developmental deviations (Marquand et al., 2019; Marquand, Rezek, Buitelaar, & Beckmann, 2016), or indices of myelination (Khelfaoui, Ibaceta‐Gonzalez, & Angulo, 2024), may therefore be required to detect mediating processes. Accordingly, data‐driven and fine‐grained neuroimaging approaches represent an important priority for future research, particularly in larger samples optimized for imaging–genetic mediation analyses.

Equally important is the timing of measurement. Genes within the NDV cluster show peak cerebellar expression during prenatal development (Hughes et al., 2023), making it plausible that polygenic risk exerts its strongest influence on early neurodevelopmental trajectories rather than on global structural volumes measured later in childhood. In this context, null mediation effects may reflect that the examined brain metrics do not explain additional variance in adolescent behavioral outcomes beyond genetic liability already indexed by the polygenic scores, rather than indicating an absence of neurobiological involvement. Longitudinal studies integrating gene‐expression data with neuroimaging across infancy, childhood, and adolescence are therefore needed to clarify the temporal dynamics of these pathways (Biagioni, Baronchelli, & Fossati, 2024).

### Limitations

Several limitations should be noted. First, because this is a population‐based cohort, the right‐skewed distribution of NDD symptoms may have attenuated associations. Selection bias is also a concern, as families with fewer difficulties were more likely to provide complete data. Replication in clinical samples will be important to establish generalizability (Hughes et al., 2023; Shakeshaft et al., 2023).

Second, although a sample of 3,270 trios is large for imaging genetics, it remains modest for detecting indirect effects, as trio models decompose variance into multiple components and often require >10,000 trios for full power (Pingault et al., 2022). Trio mediation models are also not designed to establish causality; quasi‐experimental approaches will be needed to strengthen inference about indirect genetic effects (Baud, McPeek, Chen, & Hughes, 2021), as statistically significant indirect effects may be influenced by factors beyond the anticipated environmental and biological pathways (Baud et al., 2021).

Third, the selected brain metrics may not have been sufficiently sensitive to detect mediating processes, suggesting that more refined neural phenotypes could be necessary to capture these effects.

Finally, the NDV PGS in this study was driven mainly by ADHD risk, with a smaller contribution from ASD risk, despite comparable heritability estimates (Brikell, Kuja‐Halkola, & Larsson, 2015; Tick, Bolton, Happé, Rutter, & Rijsdijk, 2015). This likely reflects the greater statistical power of ADHD GWAS and differences in discovery versus target samples. The heterogeneity of ASD phenotypes also complicates PGS prediction (Grove et al., 2019). Future work should continue to refine transdiagnostic genetic measures that balance power with specificity.

## Conclusion

In summary, this study demonstrates that direct genetic effects predominate in the transmission of neurodevelopmental disorder risk across adolescence. Maternal genetic liability for depression confers additional risk through environmentally mediated mechanisms that may involve both prenatal and postnatal influences. Within the global structural brain measures examined, we found no evidence of mediation. Nonetheless, this finding should be interpreted in the context of this study's population‐based sample and the coarse nature of the brain metrics used. These findings advance understanding of how genetic and environmental influences intersect across generations and identify maternal pathways as potential targets for prevention. Future progress will depend on richer neural phenotypes tracked longitudinally and on designs that integrate genetic nurture with measured environments to guide early interventions.

## Conflict of interest statement

No conflicts declared.

## Ethical considerations

Informed consent/assent was appropriately obtained from all participants, and where applicable, parental consent/assent was acquired in accordance with ethical guidelines. This study received ethical approval from the Medical Ethics Committee (MEC), with approval number MEC 2015–749, and the decision was made on 9 February 2016.

**Key points**

**What's known?**

- Children inherit genetic risk for neurodevelopmental disorders (NDDs), but the role of parental genetics beyond direct inheritance is less well understood.

**What's new?**

- In a large population cohort of mother–father–offspring trios, direct genetic effects were the dominant pathway linking NDD liability to adolescent behavioral problems.
- Maternal, but not paternal, depression‐related genetic liability indirectly contributed to children's difficulties through environmental pathways associated with maternal risk.
- Global brain structural measures at age 9 did not mediate the associations between genetic liability and behavioral outcomes, suggesting that gross neural phenotypes at this level of analysis are insufficient to capture the underlying pathways.

**What's relevant?**

- These findings identify maternal pathways as potential targets for support and underscore the need for mechanistically informed longitudinal studies integrating genetics, family environments, and developmental contexts.

## Supporting information


**Appendix S1.** Specification of all statistical models.
**Appendix S2.** Supplementary results.
**Table S1.** Summary of statistical models.
**Table S2.** Correlations among behavioral variables.
**Table S3.** Correlations among polygenic scores.
**Table S4a.** Latent behavioral outcomes regressed on child NDV PGS.
**Table S4b.** Latent behavioral outcomes regressed on child NDV PGS, including paternal age as a covariate.
**Table S5a.** Latent behavioral outcomes regressed on child disorder‐specific PGSs.
**Table S5b.** Latent behavioral outcomes regressed on child disorder‐specific PGSs, including paternal age as covariate.
**Table S6a.** Brain metrics regressed on child PGSs.
**Table S6b.** Brain metrics regressed on child PGSs, including paternal age as a covariate.
**Table S7a.** Trio models of NDV PGS effects on latent behavioral outcomes.
**Table S7b.** Trio models of NDV PGS effects on latent behavioral outcomes, including paternal age as a covariate.
**Table S8a.** Trio models of disorder‐specific PGS effects on latent behavioral outcomes.
**Table S8b.** Trio models of disorder‐specific PGS effects on latent behavioral outcomes, including paternal age as a covariate.
**Table S9a.** Informant‐specific trio models of MDD PGS effects on behavioral outcomes.
**Table S9b.** Informant‐specific trio models of MDD PGS effects on behavioral outcomes, including paternal age as a covariate.
**Table S10a.** NDV trio mediation effects on latent behavioral outcomes.
**Table S10b.** NDV trio mediation effects on latent behavioral outcomes, including paternal age as a covariate.

## Data Availability

The datasets generated and/or analyzed during the current study are not publicly available due to legal and ethical regulations, but may be made available upon request from the corresponding author at generationr@erasmusmc.nl, in accordance with local, national, and European Union regulations.

## References

[jcpp70184-bib-0001] Achenbach, T.M. (2010). Child behavior checklist. In Encyclopedia of cross‐cultural school psychology (pp. 546–552). New York, NY: Springer.

[jcpp70184-bib-0002] Achenbach, T.M. , Ivanova, M.Y. , Rescorla, L.A. , Turner, L.V. , & Althoff, R.R. (2016). Internalizing/externalizing problems: Review and recommendations for clinical and research applications. Journal of the American Academy of Child and Adolescent Psychiatry, 55, 647–656. 10.1016/j.jaac.2016.05.012 27453078

[jcpp70184-bib-0003] Achenbach, T.M. , & Rescorla, L.A. (2001). Manual for ASEBA school‐age forms & profiles. Burlington, VT: University of Vermont, Research Center for Children, Youth and Families.

[jcpp70184-bib-0004] Adams, M.J. , Thorp, J.G. , Jermy, B.S. , Kwong, A.S.F. , Kõiv, K. , Grotzinger, A.D. , … & Derks, E.M. (2024). Genome‐wide meta‐analysis of ascertainment and symptom structures of major depression in case‐enriched and community cohorts. Psychological Medicine, 54, 1–10. 10.1017/S0033291724001880 PMC11496230 39324397

[jcpp70184-bib-0005] Alemany, S. , Jansen, P.R. , Muetzel, R.L. , White, T. , Verhulst, F.C. , & Tiemeier, H. (2019). Common polygenic variations for psychiatric disorders and cognition in relation to brain morphology in the general pediatric population. Journal of the American Academy of Child and Adolescent Psychiatry, 58, 600–607. 10.1016/j.jaac.2018.09.443 30768412

[jcpp70184-bib-0006] Baud, A. , McPeek, S. , Chen, N. , & Hughes, K.A. (2021). Indirect genetic effects: A cross‐disciplinary perspective on empirical studies. Journal of Heredity, 113, 1–15. 10.1093/jhered/esab059 PMC8851665 34643239

[jcpp70184-bib-0007] Bauminger, N. , Solomon, M. , & Rogers, S.J. (2010). Externalizing and internalizing behaviors in ASD. Autism Research, 3, 101–112. 10.1002/aur.131 PMC5659193 20575109

[jcpp70184-bib-0008] Berg‐Nielsen, T.S. , Vika, A. , & Dahl, A.A. (2003). When adolescents disagree with their mothers: CBCL–YSR discrepancies related to maternal depression and adolescent self‐esteem. Child: Care, Health and Development, 29, 207–213. 10.1046/j.1365-2214.2003.00332.x 12752611

[jcpp70184-bib-0009] Bernard‐Bonnin, A. (2004). Maternal depression and child development. Paediatrics & Child Health, 9, 575–583. 10.1093/pch/9.8.575 PMC2724169 19680490

[jcpp70184-bib-0010] Biagioni, M. , Baronchelli, F. , & Fossati, M. (2024). Multiscale spatio‐temporal dynamics of UBE3A gene in brain physiology and neurodevelopmental disorders. Neurobiology of Disease, 201, 106669. 10.1016/j.nbd.2024.106669 39293689

[jcpp70184-bib-0011] Brikell, I. , Kuja‐Halkola, R. , & Larsson, H. (2015). Heritability of attention‐deficit hyperactivity disorder in adults. American Journal of Medical Genetics Part B: Neuropsychiatric Genetics, 168, 406–413. 10.1002/ajmg.b.32335 26129777

[jcpp70184-bib-0012] Chang, C.C. , Chow, C.C. , Tellier, L.C. , Vattikuti, S. , Purcell, S.M. , & Lee, J.J. (2015). Second‐generation PLINK: Rising to the challenge of larger and richer datasets. GigaScience, 4, 7. 10.1186/s13742-015-0047-8 PMC4342193 25722852

[jcpp70184-bib-0013] Cheesman, R. , Eilertsen, E.M. , Ahmadzadeh, Y.I. , Gjerde, L.C. , Hannigan, L.J. , Havdahl, A. , … & McAdams, T.A. (2020). How important are parents in the development of child anxiety and depression? A genomic analysis of parent–offspring trios in the Norwegian Mother, Father and Child Cohort Study (MoBa). BMC Medicine, 18, 284. 10.1186/s12916-020-01760-1 PMC7590735 33106172

[jcpp70184-bib-0014] Choi, S.W. , Mak, T.S.‐H. , & O'Reilly, P.F. (2020). Tutorial: A guide to performing polygenic risk score analyses. Nature Protocols, 15, 2759–2772. 10.1038/s41596-020-0353-1 PMC7612115 32709988

[jcpp70184-bib-0015] Connell, A.M. , & Goodman, S.H. (2002). The association between psychopathology in fathers versus mothers and children's internalizing and externalizing behavior problems: A meta‐analysis. Psychological Bulletin, 128, 746–773. 10.1037/0033-2909.128.5.746 12206193

[jcpp70184-bib-0016] Davis, E.P. , Glynn, L.M. , Schetter, C.D. , Hobel, C. , Chicz‐Demet, A. , & Sandman, C.A. (2007). Prenatal exposure to maternal depression and cortisol influences infant temperament. Journal of the American Academy of Child and Adolescent Psychiatry, 46, 737–746. 10.1097/chi.0b013e318047b775 17513986

[jcpp70184-bib-0017] Delaneau, O. , Marchini, J. , & Zagury, J.F. (2011). A linear complexity phasing method for thousands of genomes. Nature Methods, 9, 179–181. 10.1038/nmeth.1785 22138821

[jcpp70184-bib-0018] Demontis, D. , Walters, G.B. , Athanasiadis, G. , Walters, R.K. , Therrien, K. , McGrath, L.M. , & Neale, B.M. (2023). Genome‐wide analyses of ADHD identify 27 risk loci, refine the genetic architecture and implicate several cognitive domains. Nature Genetics, 55, 198–208. 10.1038/s41588-022-01285-8 PMC10914347 36702997

[jcpp70184-bib-0019] Eilertsen, E.M. , Cheesman, R. , Ayorech, Z. , Røysamb, E. , Pingault, J.‐B. , Njølstad, P.R. , … & Ystrøm, E. (2022). On the importance of parenting in externalizing disorders: An evaluation of indirect genetic effects in families. Journal of Child Psychology and Psychiatry, 63, 1098–1108. 10.1111/jcpp.13654 PMC9796091 35778910

[jcpp70184-bib-0020] Fergusson, D.M. , & Lynskey, M.T. (1993). The effects of maternal depression on child conduct disorder and attention deficit behaviours. Social Psychiatry and Psychiatric Epidemiology, 28, 116–123. 10.1007/BF00801741 8378806

[jcpp70184-bib-0021] Francés, L. , Quintero, J. , Fernández, A. , Ruiz, A. , Caules, J. , Fillon, G. , … & Soler, C.V. (2022). Current state of knowledge on the prevalence of neurodevelopmental disorders in childhood according to the DSM‐5: A systematic review in accordance with the PRISMA criteria. Child and Adolescent Psychiatry and Mental Health, 16, 1. 10.1186/s13034-022-00462-1 PMC8973738 35361232

[jcpp70184-bib-0022] Gartstein, M.A. , Bridgett, D.J. , Dishion, T.J. , & Kaufman, N.K. (2009). Depressed mood and maternal report of child behavior problems: Another look at the depression‐distortion hypothesis. Journal of Applied Developmental Psychology, 30, 149–160. 10.1016/j.appdev.2008.12.001 PMC2678740 20161323

[jcpp70184-bib-0023] Genon, S. , Eickhoff, S.B. , & Kharabian, S. (2022). Linking interindividual variability in brain structure to behaviour. Nature Reviews Neuroscience, 23, 307–318. 10.1038/s41583-022-00584-7 35365814

[jcpp70184-bib-0024] Ghatan, S. , De Vries, J. , Pingault, J. , Verhage, M.C. , Uitterlinden, A.G. , Felix, J.F. , & Tiemeier, H. (2024). Genetic nurture: Estimating the direct genetic effects of pediatric anthropometric traits. *medRxiv* 10.1101/2024.12.10.24318796 PMC12498280 40840429

[jcpp70184-bib-0025] Grove, J. , Ripke, S. , Als, T.D. , Mattheisen, M. , Walters, R.K. , Won, H. , … & Børglum, A.D. (2019). Identification of common genetic risk variants for autism spectrum disorder. Nature Genetics, 51, 431–444. 10.1038/s41588-019-0344-8 PMC6454898 30804558

[jcpp70184-bib-0026] Hegemann, L. , Eilertsen, E. , Pettersen, J.H. , Corfield, E.C. , Cheesman, R. , Frach, L. , … & Hannigan, L.J. (2025). Direct and indirect genetic effects on early neurodevelopmental traits. Journal of Child Psychology and Psychiatry, 66, 1053–1064. 10.1111/jcpp.14122 PMC12198931 39887701

[jcpp70184-bib-0027] Hoogman, M. , Muetzel, R. , Guimaraes, J.P. , Shumskaya, E. , Mennes, M. , Zwiers, M.P. , … & Franke, B. (2019). Brain imaging of the cortex in ADHD: A coordinated analysis of large‐scale clinical and population‐based samples. American Journal of Psychiatry, 176, 531–542. 10.1176/appi.ajp.2019.18091033 PMC6879185 31014101

[jcpp70184-bib-0028] Howie, B.N. , Donnelly, P. , & Marchini, J. (2009). A flexible and accurate genotype imputation method for the next generation of genome‐wide association studies. PLoS Genetics, 5, e1000529. 10.1371/journal.pgen.1000529 PMC2689936 19543373

[jcpp70184-bib-0029] Hughes, D.E. , Kunitoki, K. , Elyounssi, S. , Luo, M. , Bazer, O.M. , Hopkinson, C.E. , … & Gilman, J.M. (2023). Genetic patterning for child psychopathology is distinct from that for adults and implicates fetal cerebellar development. Nature Neuroscience, 26, 959–969. 10.1038/s41593-023-01321-8 PMC7614744 37202553

[jcpp70184-bib-0031] Ivanova, M.Y. , Achenbach, T.M. , Rescorla, L.A. , Dumenci, L. , Almqvist, F. , Bilenberg, N. , … & Verhulst, F.C. (2010). Preschool psychopathology reported by parents in 23 societies: Testing the seven‐syndrome model of the Child Behavior Checklist for ages 1.5–5. Journal of the American Academy of Child and Adolescent Psychiatry, 49, 1215–1224. 10.1016/j.jaac.2010.08.019 PMC4247330 21093771

[jcpp70184-bib-0032] Jaddoe, V.W.V. , Bakker, R. , van Duijn, C.M. , van der Heijden, A.J. , Mackenbach, J.P. , Moll, H.A. , … & Hofman, A. (2007). The generation R study biobank: A resource for epidemiological studies in children and their parents. European Journal of Epidemiology, 22, 917–923. 10.1007/s10654-007-9209-z PMC2190786 18095172

[jcpp70184-bib-0033] Khelfaoui, H. , Ibaceta‐Gonzalez, C. , & Angulo, M.C. (2024). Functional myelin in cognition and neurodevelopmental disorders. Cellular and Molecular Life Sciences, 81, 1. 10.1007/s00018-024-05222-2 PMC11016012 38615095

[jcpp70184-bib-0034] Koc, D. , Marroun, H.E. , Stricker, B.H. , Muetzel, R.L. , & Tiemeier, H. (2023). Intrauterine exposure to antidepressants or maternal depressive symptoms and offspring brain white matter trajectories from late childhood to adolescence. Biological Psychiatry: Cognitive Neuroscience and Neuroimaging, 9, 217–226. 10.1016/j.bpsc.2023.10.009 37926188

[jcpp70184-bib-0035] Kong, A. , Thorleifsson, G. , Frigge, M.L. , Vilhjalmsson, B.J. , Young, A.I. , Thorgeirsson, T.E. , … & Stefansson, K. (2018). The nature of nurture: Effects of parental genotypes. Science, 359, 424–428. 10.1126/science.aan6877 29371463

[jcpp70184-bib-0036] Kooijman, M.N. , Kruithof, C.J. , van Duijn, C.M. , Duijts, L. , Franco, O.H. , van IJzendoorn, M.H. , … & Jaddoe, V.W.V. (2016). The generation R study: Design and cohort update 2017. European Journal of Epidemiology, 31, 1243–1264. 10.1007/s10654-016-0224-9 PMC5233749 28070760

[jcpp70184-bib-0037] Krueger, R.F. , Kotov, R. , Watson, D. , Forbes, M.K. , Eaton, N.R. , Ruggero, C.J. , … & Hiatt Racer, K. (2018). Progress in achieving quantitative classification of psychopathology. World Psychiatry, 17, 282–293. 10.1002/wps.20566 PMC6172695 30229571

[jcpp70184-bib-0038] Lee, P.H. , Anttila, V. , Won, H. , Feng, Y.A. , Rosenthal, J. , Zhu, Z. , … & Smoller, J.W. (2019). Genomic relationships, novel loci, and pleiotropic mechanisms across eight psychiatric disorders. Cell, 179, 1469–1482. 10.1016/j.cell.2019.11.020 PMC7077032 31835028

[jcpp70184-bib-0039] Li, D. , Choque‐Olsson, N. , Jiao, H. , Norgren, N. , Jonsson, U. , Bölte, S. , & Tammimies, K. (2020). The influence of common polygenic risk and gene sets on social skills group training response in autism spectrum disorder. NPJ Genomic Medicine, 5, 45. 10.1038/s41525-020-00152-x PMC7550579 33083014

[jcpp70184-bib-0040] Marquand, A.F. , Kia, S.M. , Zabihi, M. , Wolfers, T. , Buitelaar, J.K. , & Beckmann, C.F. (2019). Conceptualizing mental disorders as deviations from normative functioning. Molecular Psychiatry, 24, 1415–1424. 10.1038/s41380-019-0441-1 PMC6756106 31201374

[jcpp70184-bib-0041] Marquand, A.F. , Rezek, I. , Buitelaar, J. , & Beckmann, C.F. (2016). Understanding heterogeneity in clinical cohorts using normative models: Beyond case‐control studies. Biological Psychiatry, 80, 552–561. 10.1016/j.biopsych.2015.12.023 PMC5023321 26927419

[jcpp70184-bib-0042] Medina‐Gomez, C. , Felix, J.F. , Estrada, K. , Peters, M.J. , Herrera, L. , Kruithof, C.J. , … & Rivadeneira, F. (2015). Challenges in conducting genome‐wide association studies in highly admixed multi‐ethnic populations: The Generation R Study. European Journal of Epidemiology, 30, 317–330. 10.1007/s10654-015-9998-4 PMC4385148 25762173

[jcpp70184-bib-0043] Morris‐Rosendahl, D.J. , & Crocq, M. (2020). Neurodevelopmental disorders—The history and future of a diagnostic concept. Dialogues in Clinical Neuroscience, 22, 65–72. 10.31887/DCNS.2020.22.1/macrocq PMC7365295 32699506

[jcpp70184-bib-0044] Muetzel, R.L. , Mulder, R.H. , Lamballais, S. , Cortes Hidalgo, A. , Jansen, P. , & Tiemeier, H. (2019). Frequent bullying involvement and brain morphology in children. Frontiers in Psychiatry, 10, 696. 10.3389/fpsyt.2019.00696 PMC6771170 31607968

[jcpp70184-bib-0045] Ooi, Y.P. , Rescorla, L. , Ang, R.P. , Woo, B. , & Fung, D.S.S. (2010). Identification of autism spectrum disorders using the Child Behavior Checklist in Singapore. Journal of Autism and Developmental Disorders, 41, 1147–1156. 10.1007/s10803-010-1015-x 20405192

[jcpp70184-bib-0046] Pietikäinen, J.T. , Kiviruusu, O. , Kylliäinen, A. , Pölkki, P. , Saarenpää‐Heikkilä, O. , & Paunio, T. (2019). Maternal and paternal depressive symptoms and children's emotional problems at the age of 2 and 5 years: A longitudinal study. Journal of Child Psychology and Psychiatry, 61, 195–204. 10.1111/jcpp.13126 31535379

[jcpp70184-bib-0047] Pingault, J.B. , Barkhuizen, W. , Wang, B. , Hannigan, L.J. , Eilertsen, E.M. , Corfield, E. , … & Havdahl, A. (2022). Genetic nurture versus genetic transmission of risk for ADHD traits in the Norwegian Mother, Father and Child Cohort Study. Molecular Psychiatry, 28, 1731–1738. 10.1038/s41380-022-01863-6 PMC10208953 36385167

[jcpp70184-bib-0048] Privé, F. , Albiñana, C. , Arbel, J. , Pasaniuc, B. , & Vilhjálmsson, B.J. (2023). Inferring disease architecture and predictive ability with LDpred2‐auto. American Journal of Human Genetics, 110, 2042–2055. 10.1016/j.ajhg.2023.10.010 PMC10716363 37944514

[jcpp70184-bib-0049] Privé, F. , Arbel, J. , & Vilhjálmsson, B.J. (2020). LDpred2: Better, faster, stronger. Bioinformatics, 36, 5424–5431. 10.1093/bioinformatics/btaa1029 PMC8016455 33326037

[jcpp70184-bib-0050] Purcell, S. , Neale, B. , Todd‐Brown, K. , Thomas, L. , Ferreira, M.A.R. , Bender, D. , … & Sham, P.C. (2007). PLINK: A tool set for whole‐genome association and population‐based linkage analyses. American Journal of Human Genetics, 81, 559–575. 10.1086/519795 PMC1950838 17701901

[jcpp70184-bib-0051] Rosseel, Y. (2012). lavaan: An R package for structural equation modeling. Journal of Statistical Software, 48, 1–36.

[jcpp70184-bib-0052] Scandurra, V. , Gialloreti, L.E. , Barbanera, F. , Scordo, M.R. , Pierini, A. , & Canitano, R. (2019). Neurodevelopmental disorders and adaptive functions: A study of children with autism spectrum disorders (ASD) and/or attention deficit and hyperactivity disorder (ADHD). Frontiers in Psychiatry, 10, 673. 10.3389/fpsyt.2019.00673 PMC6737073 31551839

[jcpp70184-bib-0053] Shakeshaft, A. , Martin, J. , Dennison, C.A. , Riglin, L. , Lewis, C.M. , O'Donovan, M.C. , & Walters, J.T.R. (2023). Estimating the impact of transmitted and non‐transmitted psychiatric and neurodevelopmental polygenic scores on youth emotional problems. Molecular Psychiatry, 29, 238–246. 10.1038/s41380-023-02319-1 PMC11116100 37990052

[jcpp70184-bib-0054] Shi, Y. , Sprooten, E. , Mulders, P. , Vrijsen, J. , Bralten, J. , Demontis, D. , … & Mota, N.R. (2023). Multi‐polygenic scores in psychiatry: From disorder‐specific to transdiagnostic perspectives. American Journal of Medical Genetics. Part B, Neuropsychiatric Genetics, 195, 1–12. 10.1002/ajmg.b.32951 PMC10803201 37334623

[jcpp70184-bib-0055] Thapar, A. , Cooper, M. , & Rutter, M. (2017). Neurodevelopmental disorders. The Lancet Psychiatry, 4, 339–346. 10.1016/S2215-0366(16)30376-5 27979720

[jcpp70184-bib-0056] Tick, B. , Bolton, P. , Happé, F. , Rutter, M. , & Rijsdijk, F. (2015). Heritability of autism spectrum disorders: A meta‐analysis of twin studies. Journal of Child Psychology and Psychiatry, 57, 585–595. 10.1111/jcpp.12499 PMC4996332 26709141

[jcpp70184-bib-0057] Van Rooij, D. , Anagnostou, E. , Arango, C. , Auzias, G. , Behrmann, M. , Busatto, G.F. , … & Buitelaar, J.K. (2017). Cortical and subcortical brain morphometry differences between patients with autism spectrum disorder and healthy individuals across the lifespan: Results from the ENIGMA ASD working group. American Journal of Psychiatry, 175, 359–369. 10.1176/appi.ajp.2017.17010100 PMC6546164 29145754

[jcpp70184-bib-0058] Vieira, S. , Bolton, T.A.W. , Schöttner, M. , Baecker, L. , Marquand, A.F. , Mechelli, A. , … & Sethi, A. (2024). Multivariate brain‐behaviour associations in psychiatric disorders. Translational Psychiatry, 14, 1. 10.1038/s41398-024-02954-4 PMC11144193 38824172

[jcpp70184-bib-0059] Voronin, I. , Ouellet‐Morin, I. , Petitclerc, A. , Morneau‐Vaillancourt, G. , Brendgen, M. , Dione, G. , … & Boivin, M. (2024). Intergenerational transmission of genetic risk for hyperactivity and inattention: Direct genetic transmission or genetic nurture? JCPP Advances, 4, e12222. 10.1002/jcv2.12222 PMC11143957 38827976

[jcpp70184-bib-0060] White, T. , Muetzel, R.L. , Marroun, H.E. , Blanken, L.M.E. , Jansen, P.W. , Bolhuis, K. , … & Tiemeier, H. (2017). Paediatric population neuroimaging and the generation R study: The second wave. European Journal of Epidemiology, 33, 99–125. 10.1007/s10654-017-0319-y PMC5803295 29064008

