# Supplementary material for: Disentangling direct and indirect genetic pathways to neurodevelopmental risk: brain structure and behavior in a population‐based parent–offspring trio study
Source: J Child Psychol Psychiatry. 2026 Jun 10;67(11):1831–43. doi: 10.1111/jcpp.70184 (PMC13647101; doi:10.1111/jcpp.70184)
Supplement: Supplementary file 1 [file JCPP-67-1831-s001.docx]

**Appendix S1. Specification of All Statistical Models**

**Purpose:**

This Supporting Information section provides a complete and explicit overview of all statistical models estimated in the study. For each model class, we specify the variables included, the pathways estimated, the outcome operationalization, covariates, and model constraints. This Appendix is intended to allow full reconstruction of the analytic strategy while keeping the main manuscript concise.

**1. Overview of Model Classes**

- **Model class 1:** Genetic association models
- **Model class 2:** Trio models (direct vs indirect genetic effects)
- **Model class 3:** Trio mediation models (brain structure as mediator)

**2. Association Models**

**Overview**

Association models were estimated to quantify total (unpartitioned) associations between polygenic risk, behavioral outcomes, and neuroimaging phenotypes prior to decomposition into direct and indirect genetic effects (Figure 1A). All models included a single PGS type per model (NDV, ADHD, ASD, or MDD), modeled separately. Behavioral and neuroimaging outcomes were analyzed in separate models. Missing data were handled using full-information maximum likelihood (FIML), and false discovery rate (FDR) correction was applied within predefined test families.

**2.1 Behavioral Association Models (Primary Analyses)**

For each combination of PGS type and behavioral domain, a separate structural equation model (SEM) was estimated.

**Model structure**

Each behavioral domain was modeled as a latent variable representing shared variance between parent-reported and self-reported symptoms.

The following paths were estimated in each model:

**Measurement paths**

- The latent behavioral factor was indicated by:
  - CBCL domain score (parent-report)
  - YSR domain score (self-report)
- The YSR loading was fixed to 1 for scale identification.
- The CBCL loading was freely estimated.
- Residual variances of both indicators were freely estimated.

**Structural path**

- The latent behavioral factor was regressed on the child polygenic score.

**Predictor**

- Child PGS (NDV, ADHD, ASD, or MDD: modeled separately)

**Behavioral domains**

- Internalizing problems
- Externalizing problems
- Attention problems
- Social problems

**Covariates**

- Child age at assessment
- Maternal age at conception
- Child sex (residualized prior to modeling)

**Estimation**

- SEM with robust maximum likelihood (MLR)
- FIML for missing data

**Multiple testing**

- NDV PGS: FDR correction across 4 tests (one per behavioral domain)
- Disorder-specific PGSs (ADHD, ASD, MDD): FDR correction across 12 tests (3 PGSs × 4 domains)

**2.2 Neuroimaging Association Models (Primary Analyses)**

For each combination of PGS type and brain metric, a separate association model was estimated.

**Model structure**

Each model estimated a single regression path:

- Brain metric regressed on the child polygenic score

**Predictors**

- Child PGS (NDV, ADHD, ASD, or MDD; modeled separately)

**Outcomes**

- Cortical surface area
- Subcortical gray matter volume
- Cerebellar cortical volume

Each brain metric was modeled as a single observed outcome in a separate model.

**Covariates**

- Child age at assessment
- Child sex
- Intracranial volume (ICV)

**Estimation**

- SEM framework with observed outcomes
- Robust maximum likelihood estimator (MLR)
- Full-information maximum likelihood (FIML)

**Multiple testing**

- NDV PGS: FDR correction across 3 tests (one per brain metric)
- Disorder-specific PGSs: FDR correction across 9 tests (3 PGSs × 3 metrics)

**2.3 Informant-Specific Behavioral Models**

To facilitate interpretation of potential informant-related patterns, behavioral association models were re-estimated using single-informant observed outcomes, replacing the latent factor with:

- CBCL domain score only, or
- YSR domain score only.

The same predictors, covariates, and estimation procedures as in the primary behavioral association models were used.

These informant-specific models:

- Were conducted for completeness only
- Were not part of the primary hypothesis-testing framework
- Were excluded from all FDR-corrected test families
- Are reported exclusively in the Supporting Information

**3. Trio Models**

**Overview**

Trio models were estimated to decompose total genetic associations into direct child genetic effects and indirect parental genetic effects (genetic nurture). These models explicitly account for genetic transmission within families and correspond to Figure 1B. All models were estimated separately by polygenic score (PGS) type (NDV, ADHD, ASD, or MDD).

**3.1 Behavioral Trio Models (Primary Analyses)**

For each behavioral domain, one structural equation model (SEM) was estimated per PGS type. Each model included multiple family-member PGSs by design (child, mother, father), but no model included more than one PGS type simultaneously.

**Model structure**

Within each trio model, the following paths were estimated:

**Genetic transmission paths**

- The child PGS was regressed on the maternal PGS.
- The child PGS was regressed on the paternal PGS.

This specification accounts for inherited genetic effects and ensures that associations between parental PGSs and the child outcome represent indirect (environmentally mediated) pathways rather than shared genetic transmission.

**Direct and indirect effect paths**

- The behavioral outcome was regressed on the child PGS (direct genetic effect).
- The behavioral outcome was regressed on the maternal PGS (maternal indirect effect).
- The behavioral outcome was regressed on the paternal PGS (paternal indirect effect).

**Covariance path**

- Maternal and paternal PGSs were allowed to covary to account for assortative mating.

**Predictors (modeled jointly within each trio model)**

- Child PGS
- Maternal PGS
- Paternal PGS

**Outcome**

Behavioral outcomes were modeled as latent factors capturing shared variance between parent- and self-reported symptoms.

The latent behavioral factor was indicated by:

- CBCL domain score (parent-report)
- YSR domain score (self-report)

**Domains:**

- Internalizing problems
- Externalizing problems
- Attention problems
- Social problems

**Latent-factor specification:**

- YSR loading fixed to 1 for scale identification
- CBCL loading freely estimated
- Indicator residual variances freely estimated

**Covariates**

- Child age at assessment
- Maternal age at conception
- Child sex (residualized prior to modeling)

**Estimation**

- SEM with robust maximum likelihood estimator (MLR)
- Full-information maximum likelihood (FIML) for missing data

**Multiple testing (primary trio models)**

False discovery rate (FDR) correction was applied within predefined test families, separately for each genetic pathway, rather than across all parameter estimates within a model or across all trio models jointly.

Specifically, FDR correction was applied as follows:

For the direct child genetic pathway:

- NDV PGS: 4 tests (one per behavioral domain)
- Disorder-specific PGSs (ADHD, ASD, MDD): 12 tests (3 PGSs × 4 domains)

For the maternal indirect genetic pathway:

- NDV PGS: 4 tests
- Disorder-specific PGSs: 12 tests

For the paternal indirect genetic pathway:

- NDV PGS: 4 tests
- Disorder-specific PGSs: 12 tests

Thus, direct and indirect genetic effects were evaluated against separate multiple-testing thresholds, consistent with their distinct theoretical interpretation in a trio design. This strategy reflects the a priori distinction between transdiagnostic and disorder-specific genetic pathways and balances control of type I error with adequate power to detect indirect genetic effects.

**3.2 Informant-Specific Trio Models (Targeted Follow-up Analyses)**

To directly evaluate potential rater-related influences on the observed maternal indirect effects of the MDD PGS, trio models were re-estimated using single-informant observed outcomes rather than latent factors.

Specifically, separate models were estimated using:

- CBCL domain scores only (parent-report), and
- YSR domain scores only (self-report),

for each behavioral domain (internalizing, externalizing, attention problems, social problems).

The genetic structure of the models remained identical to the primary trio models:

- Child PGS regressed on maternal and paternal PGSs
- Behavioral outcome regressed on child, maternal, and paternal PGSs
- Maternal–paternal PGS covariance freely estimated

Covariates and estimation procedures were identical to those used in the primary trio models.

**Multiple testing (informant-specific follow-up)**

These targeted follow-up analyses were restricted to the MDD PGS and comprised:

- Eight comparisons (2 informants × 4 behavioral domains)

FDR correction was applied across these eight tests.

Informant-specific trio models for other PGSs (NDV, ADHD, ASD) were also estimated for completeness, but:

- Were not part of the primary hypothesis-testing framework
- Were not included in any FDR-corrected test family
- Are reported exclusively in the Supporting Information

**4. Trio Mediation Models**

**Overview**

Trio mediation models were estimated to examine whether childhood brain structure statistically mediates direct or indirect genetic effects of polygenic risk on adolescent behavioral outcomes. These models extend the trio transmission models described in Section 3 by introducing a single neuroimaging variable as a mediator between polygenic scores and behavioral outcomes and correspond to Figure 1C. All mediation analyses were conducted exclusively for the NDV polygenic score, given the absence of robust brain–PGS associations for disorder-specific scores in preceding analyses.

**4.1 Primary Mediation Models (NDV PGS)**

For each behavioral domain, separate mediation models were estimated, such that one neuroimaging mediator was included per model.

Relative to the behavioral trio models described in Section 3.1, the mediation models added a brain mediator while retaining the full trio structure. All genetic transmission paths, covariates, latent outcome specification, and parental PGS covariance remained unchanged.

**Model structure**

Within each mediation model, the following paths were estimated:

**Genetic transmission paths (unchanged from trio models)**

- The child NDV PGS was regressed on the maternal NDV PGS.
- The child NDV PGS was regressed on the paternal NDV PGS.

**Direct and indirect effect paths (unchanged)**

- The behavioral outcome was regressed on the child NDV PGS (direct genetic effect).
- The behavioral outcome was regressed on the maternal NDV PGS (maternal indirect effect).
- The behavioral outcome was regressed on the paternal NDV PGS (paternal indirect effect).

**Mediator paths (new in mediation models)**

- The brain metric was regressed on the child NDV PGS.
- The brain metric was regressed on the maternal NDV PGS.
- The brain metric was regressed on the paternal NDV PGS.
- The behavioral outcome was regressed on the brain metric.

Together, these paths define three mediation pathways per model:
child pathway, maternal pathway, and paternal pathway.

Mediation effects were defined as the product of the corresponding PGS-to-brain path and the brain-to-outcome path.

**Covariance path**

- Maternal and paternal NDV PGSs were allowed to covary, as in the trio models.

**Predictors (modeled jointly)**

- Child NDV PGS
- Maternal NDV PGS
- Paternal NDV PGS

**Mediator**

One neuroimaging variable per model:

- Cortical surface area
- Subcortical gray matter volume
- Cerebellar cortical volume

**Outcome**

Behavioral outcomes were modeled as latent factors capturing shared variance between parent- and self-reported symptoms.

**The latent behavioral factor was indicated by:**

- CBCL domain score (parent-report)
- YSR domain score (self-report)

**Domains:**

- Internalizing problems
- Externalizing problems
- Attention problems
- Social problems

Latent-factor specification matched earlier association and trio models, with the YSR loading fixed to 1, the CBCL loading freely estimated, and indicator residual variances freely estimated.

**Covariates**

- Child age at assessment
- Maternal age at conception
- Child sex (residualized prior to modeling)
- Intracranial volume, included in all regression paths involving brain metrics

**Estimation**

- Sem with robust maximum likelihood estimator (MLR)
- Full-information maximum likelihood (FIML) for missing data

**Hypothesis testing and multiple testing correction**

Although full mediation models were estimated, statistical inference focused exclusively on the mediation (indirect) parameters. Direct genetic effects, transmission paths, and covariances were estimated for model completeness but were not part of the mediation hypothesis tests.

False discovery rate (FDR) correction was applied separately within each genetic pathway, reflecting the a priori distinction between:

- Child pathway
- Maternal pathway
- Paternal pathway

For each pathway, mediation effects were evaluated across:

- Twelve tests, corresponding to four behavioral domains multiplied by three brain metrics.

Thus, mediation effects for each genetic pathway were evaluated against pathway-specific multiple-testing thresholds.

**4.2 Informant-Specific Mediation Models (Follow-up Analyses)**

To further contextualize the primary mediation findings and assess potential informant-related patterns, mediation models were re-estimated using single-informant observed outcomes rather than latent behavioral factors.

Specifically, models were estimated using:

- CBCL domain scores only (parent-report), and
- YSR domain scores only (self-report),

for each behavioral domain and brain metric.

The genetic structure of the models remained identical to the primary mediation models:

- Child NDV PGS regressed on maternal and paternal NDV PGSs
- Behavioral outcome regressed on child, maternal, and paternal NDV PGSs
- Brain metric regressed on child, maternal, and paternal NDV PGSs
- Behavioral outcome regressed on the brain metric
- Maternal–paternal NDV PGS covariance freely estimated

Covariates and estimation procedures were identical to those used in the primary mediation models.

**Multiple testing**

For each genetic pathway, these follow-up analyses comprised:

- Twenty-four mediation tests, corresponding to two informants multiplied by four behavioral domains multiplied by three brain metrics.

FDR correction was applied within each pathway-specific informant family.

Results from these models are reported in the Supporting Information and were used to contextualize the primary mediation findings rather than for confirmatory inference.

**5. Sensitivity Analysis: Paternal Age**

In an additional sensitivity analysis, paternal age was included as a covariate alongside maternal age in all models. This was done to assess the potential impact of including paternal age on the overall model structure and to ensure robustness of the findings. All models (primary, trio, and mediation) were re-estimated with paternal age as an additional covariate, while retaining the same covariates as in the original models.

**6.** **Table S1. Summary of Statistical Models**

| **Model Class** | **Purpose** | **Genetic Predictors** | **Outcomes** | **Mediator(s)** | **Informant Structure** | **Covariates** | **N Tests** | **FDR Family** |
| --- | --- | --- | --- | --- | --- | --- | --- | --- |
| Association (Behavior) | Total genetic associations | Child PGS (NDV / ADHD / ASD / MDD; modeled separately) | Behavioral domains (internalizing, externalizing, attention, social) | – | Latent CBCL–YSR factor | Child age, maternal age, sex | NDV: 4; disorder-specific PGSs: 12 | Per PGS family |
| Association (Brain) | PGS–brain associations | Child PGS (NDV / ADHD / ASD / MDD; modeled separately) | Brain metrics (cortical surface area, subcortical GM, cerebellum) | – | – | Child age, sex, intracranial volume | NDV: 3; disorder-specific PGSs: 9 | Per PGS family |
| Trio (Primary) | Direct vs. indirect genetic effects | Child, maternal, and paternal PGS (modeled jointly within each PGS family) | Behavioral domains | – | Latent CBCL–YSR factor | Child age, maternal age, sex | NDV: 4; disorder-specific PGSs: 12 | Per PGS family x genetic pathway |
| Trio (Informant-specific) | Rater-related follow-up (MDD) | Child, maternal, and paternal MDD PGS | Behavioral domains | – | CBCL-only or YSR-only | Child age, maternal age, sex | 8 | Per pathway (MDD only) |
| Trio Mediation (Primary) | Brain-mediated genetic effects | Child, maternal, and paternal NDV PGS | Behavioral domains | Cortical surface area, subcortical GM, cerebellum | Latent CBCL–YSR factor | Child age, maternal age, sex, intracranial volume | 12 per pathway | Per genetic pathway (NDV only) |
| Trio Mediation (Informant-specific) | Informant-specific follow-up (NDV) | Child, maternal, and paternal NDV PGS | Behavioral domains | Cortical surface area, subcortical GM, cerebellum | CBCL-only or YSR-only | Child age, maternal age, sex, intracranial volume | 24 per pathway | Per genetic pathway (NDV only) |

**Notes.**

- All models were estimated using structural equation modeling with robust maximum likelihood estimation and full-information maximum likelihood to handle missing data.
- False discovery rate (FDR) correction was applied within predefined test families defined jointly by polygenic score (PGS) family and genetic pathway. Specifically, separate FDR procedures were applied for each PGS family (NDV vs. disorder-specific PGSs) and within each family separately for direct child effects, maternal indirect effects, and paternal indirect effects. Thus, multiple testing correction was not applied across pathways or across PGS families jointly.
- Disorder-specific polygenic scores were not examined in mediation models due to the absence of robust PGS–brain associations.
- Informant-specific models were conducted either as targeted hypothesis tests (maternal MDD indirect effects) or as follow-up contextual analyses (NDV mediation) and are fully reported in the Supporting Information.

**Appendix S2.** **Supplementary Results**

**Table S2.** **Correlations Among Behavioral Variables**

| **Variable 1** | **Variable 2** | **r** | **CI** | **p** |
| --- | --- | --- | --- | --- |
| YSR Social | YSR Attention | 0.55 | [.52, .57] | <0.001 |
| YSR Social | YSR Internalizing | 0.63 | [.61, .64] | <0.001 |
| YSR Social | YSR Externalizing | 0.55 | [.53, .57] | <0.001 |
| YSR Social | CBCL Social | 0.41 | [.38, .43] | <0.001 |
| YSR Social | CBCL Attention | 0.30 | [.28, .33] | <0.001 |
| YSR Social | CBCL Internalizing | 0.33 | [.31, .36] | <0.001 |
| YSR Social | CBCL Externalizing | 0.28 | [.25, .31] | <0.001 |
| YSR Attention | YSR Internalizing | 0.47 | [.44, .49] | <0.001 |
| YSR Attention | YSR Externalizing | 0.59 | [.57, .61] | <0.001 |
| YSR Attention | CBCL Social | 0.30 | [.28, .33] | <0.001 |
| YSR Attention | CBCL Attention | 0.55 | [.53, .57] | <0.001 |
| YSR Attention | CBCL Internalizing | 0.28 | [.25, .31] | <0.001 |
| YSR Attention | CBCL Externalizing | 0.34 | [.32, .37] | <0.001 |
| YSR Internalizing | YSR Externalizing | 0.48 | [.46, .50] | <0.001 |
| YSR Internalizing | CBCL Social | 0.36 | [.33, .38] | <0.001 |
| YSR Internalizing | CBCL Attention | 0.23 | [.20, .26] | <0.001 |
| YSR Internalizing | CBCL Internalizing | 0.52 | [.50, .55] | <0.001 |
| YSR Internalizing | CBCL Externalizing | 0.26 | [.23, .29] | <0.001 |
| YSR Externalizing | CBCL Social | 0.33 | [.30, .35] | <0.001 |
| YSR Externalizing | CBCL Attention | 0.39 | [.37, .42] | <0.001 |
| YSR Externalizing | CBCL Internalizing | 0.27 | [.24, .30] | <0.001 |
| YSR Externalizing | CBCL Externalizing | 0.55 | [.53, .57] | <0.001 |
| CBCL Social | CBCL Attention | 0.56 | [.54, .58] | <0.001 |
| CBCL Social | CBCL Internalizing | 0.64 | [.62, .66] | <0.001 |
| CBCL Social | CBCL Externalizing | 0.60 | [.58, .62] | <0.001 |
| CBCL Attention | CBCL Internalizing | 0.48 | [.45, .50] | <0.001 |
| CBCL Attention | CBCL Externalizing | 0.61 | [.59, .63] | <0.001 |
| CBCL Internalizing | CBCL Externalizing | 0.54 | [.52, .56] | <0.001 |

**Table S3.** **Correlations Among Polygenic Scores**

| **Predictor Trait** | **Predictor Type** | **Outcome Trait** | **Outcome Type** | **r** | **CI Lower** | **CI Upper** | **p** |
| --- | --- | --- | --- | --- | --- | --- | --- |
| ADHD | child | ASD | child | 0.29 | 0.26 | 0.32 | <0.001 |
| ADHD | child | MDD | child | 0.27 | 0.24 | 0.30 | <0.001 |
| ADHD | child | NDV | child | 0.66 | 0.64 | 0.68 | <0.001 |
| ADHD | child | ADHD | mother | 0.51 | 0.49 | 0.54 | <0.001 |
| ADHD | child | ASD | mother | 0.14 | 0.11 | 0.18 | <0.001 |
| ADHD | child | MDD | mother | 0.15 | 0.11 | 0.18 | <0.001 |
| ADHD | child | NDV | mother | 0.36 | 0.33 | 0.39 | <0.001 |
| ADHD | child | ADHD | father | 0.51 | 0.48 | 0.53 | <0.001 |
| ADHD | child | ASD | father | 0.12 | 0.09 | 0.15 | <0.001 |
| ADHD | child | MDD | father | 0.15 | 0.12 | 0.18 | <0.001 |
| ADHD | child | NDV | father | 0.33 | 0.30 | 0.36 | <0.001 |
| ASD | child | ADHD | child | 0.29 | 0.26 | 0.32 | <0.001 |
| ASD | child | MDD | child | 0.13 | 0.10 | 0.16 | <0.001 |
| ASD | child | NDV | child | 0.54 | 0.51 | 0.56 | <0.001 |
| ASD | child | ADHD | mother | 0.13 | 0.10 | 0.17 | <0.001 |
| ASD | child | ASD | mother | 0.48 | 0.45 | 0.51 | <0.001 |
| ASD | child | MDD | mother | 0.07 | 0.04 | 0.11 | <0.001 |
| ASD | child | NDV | mother | 0.27 | 0.24 | 0.31 | <0.001 |
| ASD | child | ADHD | father | 0.15 | 0.11 | 0.18 | <0.001 |
| ASD | child | ASD | father | 0.49 | 0.47 | 0.52 | <0.001 |
| ASD | child | MDD | father | 0.06 | 0.03 | 0.10 | <0.001 |
| ASD | child | NDV | father | 0.25 | 0.21 | 0.28 | <0.001 |
| MDD | child | ADHD | child | 0.27 | 0.24 | 0.30 | <0.001 |
| MDD | child | ASD | child | 0.13 | 0.10 | 0.16 | <0.001 |
| MDD | child | NDV | child | 0.44 | 0.41 | 0.47 | <0.001 |
| MDD | child | ADHD | mother | 0.13 | 0.09 | 0.16 | <0.001 |
| MDD | child | ASD | mother | 0.07 | 0.04 | 0.11 | <0.001 |
| MDD | child | MDD | mother | 0.49 | 0.47 | 0.52 | <0.001 |
| MDD | child | NDV | mother | 0.23 | 0.20 | 0.26 | <0.001 |
| MDD | child | ADHD | father | 0.15 | 0.12 | 0.19 | <0.001 |
| MDD | child | ASD | father | 0.06 | 0.03 | 0.09 | 0.001 |
| MDD | child | MDD | father | 0.49 | 0.47 | 0.52 | <0.001 |
| MDD | child | NDV | father | 0.23 | 0.19 | 0.26 | <0.001 |
| NDV | child | ADHD | child | 0.66 | 0.64 | 0.68 | <0.001 |
| NDV | child | ASD | child | 0.54 | 0.51 | 0.56 | <0.001 |
| NDV | child | MDD | child | 0.44 | 0.41 | 0.47 | <0.001 |
| NDV | child | ADHD | mother | 0.33 | 0.30 | 0.36 | <0.001 |
| NDV | child | ASD | mother | 0.26 | 0.23 | 0.29 | <0.001 |
| NDV | child | MDD | mother | 0.23 | 0.20 | 0.26 | <0.001 |
| NDV | child | NDV | mother | 0.51 | 0.49 | 0.54 | <0.001 |
| NDV | child | ADHD | father | 0.33 | 0.30 | 0.36 | <0.001 |
| NDV | child | ASD | father | 0.25 | 0.22 | 0.28 | <0.001 |
| NDV | child | MDD | father | 0.21 | 0.18 | 0.24 | <0.001 |
| NDV | child | NDV | father | 0.49 | 0.46 | 0.51 | <0.001 |
| ADHD | mother | ADHD | child | 0.51 | 0.49 | 0.54 | <0.001 |
| ADHD | mother | ASD | child | 0.13 | 0.10 | 0.17 | <0.001 |
| ADHD | mother | MDD | child | 0.13 | 0.09 | 0.16 | <0.001 |
| ADHD | mother | NDV | child | 0.33 | 0.30 | 0.36 | <0.001 |
| ADHD | mother | ASD | mother | 0.28 | 0.25 | 0.32 | <0.001 |
| ADHD | mother | MDD | mother | 0.26 | 0.23 | 0.30 | <0.001 |
| ADHD | mother | NDV | mother | 0.65 | 0.63 | 0.67 | <0.001 |
| ADHD | mother | ADHD | father | 0.06 | 0.02 | 0.09 | 0.001 |
| ADHD | mother | ASD | father | -0.03 | -0.07 | 0.00 | 0.056 |
| ADHD | mother | MDD | father | 0.00 | -0.04 | 0.03 | 0.838 |
| ADHD | mother | NDV | father | 0.02 | -0.01 | 0.06 | 0.205 |
| ASD | mother | ADHD | child | 0.14 | 0.11 | 0.18 | <0.001 |
| ASD | mother | ASD | child | 0.48 | 0.45 | 0.51 | <0.001 |
| ASD | mother | MDD | child | 0.07 | 0.04 | 0.11 | <0.001 |
| ASD | mother | NDV | child | 0.26 | 0.23 | 0.29 | <0.001 |
| ASD | mother | ADHD | mother | 0.28 | 0.25 | 0.32 | <0.001 |
| ASD | mother | MDD | mother | 0.16 | 0.13 | 0.19 | <0.001 |
| ASD | mother | NDV | mother | 0.53 | 0.51 | 0.56 | <0.001 |
| ASD | mother | ADHD | father | 0.00 | -0.03 | 0.04 | 0.857 |
| ASD | mother | ASD | father | -0.01 | -0.05 | 0.02 | 0.428 |
| ASD | mother | MDD | father | -0.02 | -0.05 | 0.02 | 0.322 |
| ASD | mother | NDV | father | -0.01 | -0.04 | 0.03 | 0.671 |
| MDD | mother | ADHD | child | 0.15 | 0.11 | 0.18 | <0.001 |
| MDD | mother | ASD | child | 0.07 | 0.04 | 0.11 | <0.001 |
| MDD | mother | MDD | child | 0.49 | 0.47 | 0.52 | <0.001 |
| MDD | mother | NDV | child | 0.23 | 0.20 | 0.26 | <0.001 |
| MDD | mother | ADHD | mother | 0.26 | 0.23 | 0.30 | <0.001 |
| MDD | mother | ASD | mother | 0.16 | 0.13 | 0.19 | <0.001 |
| MDD | mother | NDV | mother | 0.46 | 0.44 | 0.49 | <0.001 |
| MDD | mother | ADHD | father | 0.02 | -0.01 | 0.06 | 0.187 |
| MDD | mother | ASD | father | -0.02 | -0.05 | 0.02 | 0.308 |
| MDD | mother | MDD | father | 0.00 | -0.04 | 0.03 | 0.826 |
| MDD | mother | NDV | father | 0.01 | -0.03 | 0.04 | 0.599 |
| NDV | mother | ADHD | child | 0.36 | 0.33 | 0.39 | <0.001 |
| NDV | mother | ASD | child | 0.27 | 0.24 | 0.31 | <0.001 |
| NDV | mother | MDD | child | 0.23 | 0.20 | 0.26 | <0.001 |
| NDV | mother | NDV | child | 0.51 | 0.49 | 0.54 | <0.001 |
| NDV | mother | ADHD | mother | 0.65 | 0.63 | 0.67 | <0.001 |
| NDV | mother | ASD | mother | 0.53 | 0.51 | 0.56 | <0.001 |
| NDV | mother | MDD | mother | 0.46 | 0.44 | 0.49 | <0.001 |
| NDV | mother | ADHD | father | 0.05 | 0.02 | 0.08 | 0.004 |
| NDV | mother | ASD | father | -0.01 | -0.04 | 0.02 | 0.557 |
| NDV | mother | MDD | father | -0.01 | -0.04 | 0.02 | 0.589 |
| NDV | mother | NDV | father | 0.02 | -0.01 | 0.06 | 0.204 |
| ADHD | father | ADHD | child | 0.51 | 0.48 | 0.53 | <0.001 |
| ADHD | father | ASD | child | 0.15 | 0.11 | 0.18 | <0.001 |
| ADHD | father | MDD | child | 0.15 | 0.12 | 0.19 | <0.001 |
| ADHD | father | NDV | child | 0.33 | 0.30 | 0.36 | <0.001 |
| ADHD | father | ADHD | mother | 0.06 | 0.02 | 0.09 | 0.001 |
| ADHD | father | ASD | mother | 0.00 | -0.03 | 0.04 | 0.857 |
| ADHD | father | MDD | mother | 0.02 | -0.01 | 0.06 | 0.187 |
| ADHD | father | NDV | mother | 0.05 | 0.02 | 0.08 | 0.004 |
| ADHD | father | ASD | father | 0.27 | 0.24 | 0.30 | <0.001 |
| ADHD | father | MDD | father | 0.30 | 0.27 | 0.33 | <0.001 |
| ADHD | father | NDV | father | 0.65 | 0.63 | 0.67 | <0.001 |
| ASD | father | ADHD | child | 0.12 | 0.09 | 0.15 | <0.001 |
| ASD | father | ASD | child | 0.49 | 0.47 | 0.52 | <0.001 |
| ASD | father | MDD | child | 0.06 | 0.03 | 0.09 | 0.001 |
| ASD | father | NDV | child | 0.25 | 0.22 | 0.28 | <0.001 |
| ASD | father | ADHD | mother | -0.03 | -0.07 | 0.00 | 0.056 |
| ASD | father | ASD | mother | -0.01 | -0.05 | 0.02 | 0.428 |
| ASD | father | MDD | mother | -0.02 | -0.05 | 0.02 | 0.308 |
| ASD | father | NDV | mother | -0.01 | -0.04 | 0.02 | 0.557 |
| ASD | father | ADHD | father | 0.27 | 0.24 | 0.30 | <0.001 |
| ASD | father | MDD | father | 0.13 | 0.10 | 0.17 | <0.001 |
| ASD | father | NDV | father | 0.51 | 0.48 | 0.54 | <0.001 |
| MDD | father | ADHD | child | 0.15 | 0.12 | 0.18 | <0.001 |
| MDD | father | ASD | child | 0.06 | 0.03 | 0.10 | <0.001 |
| MDD | father | MDD | child | 0.49 | 0.47 | 0.52 | <0.001 |
| MDD | father | NDV | child | 0.21 | 0.18 | 0.24 | <0.001 |
| MDD | father | ADHD | mother | 0.00 | -0.04 | 0.03 | 0.838 |
| MDD | father | ASD | mother | -0.02 | -0.05 | 0.02 | 0.322 |
| MDD | father | MDD | mother | 0.00 | -0.04 | 0.03 | 0.826 |
| MDD | father | NDV | mother | -0.01 | -0.04 | 0.02 | 0.589 |
| MDD | father | ADHD | father | 0.30 | 0.27 | 0.33 | <0.001 |
| MDD | father | ASD | father | 0.13 | 0.10 | 0.17 | <0.001 |
| MDD | father | NDV | father | 0.46 | 0.43 | 0.49 | <0.001 |
| NDV | father | ADHD | child | 0.33 | 0.30 | 0.36 | <0.001 |
| NDV | father | ASD | child | 0.25 | 0.21 | 0.28 | <0.001 |
| NDV | father | MDD | child | 0.23 | 0.19 | 0.26 | <0.001 |
| NDV | father | NDV | child | 0.49 | 0.46 | 0.51 | <0.001 |
| NDV | father | ADHD | mother | 0.02 | -0.01 | 0.06 | 0.205 |
| NDV | father | ASD | mother | -0.01 | -0.04 | 0.03 | 0.671 |
| NDV | father | MDD | mother | 0.01 | -0.03 | 0.04 | 0.599 |
| NDV | father | NDV | mother | 0.02 | -0.01 | 0.06 | 0.204 |
| NDV | father | ADHD | father | 0.65 | 0.63 | 0.67 | <0.001 |
| NDV | father | ASD | father | 0.51 | 0.48 | 0.54 | <0.001 |
| NDV | father | MDD | father | 0.46 | 0.43 | 0.49 | <0.001 |

**Table S4a.** **Latent Behavioral Outcomes Regressed on Child NDV PGS**

| **PGS** | **Domain** | **Estimate** | **SE** | **z** | **p** | **CI Lower** | **CI Upper** | **Std.Est** |
| --- | --- | --- | --- | --- | --- | --- | --- | --- |
| NDV Child | Social | 0.248 | 0.094 | 2.638 | 0.008 | 0.064 | 0.433 | 0.122 |
| NDV Child | Attention | 0.677 | 0.132 | 5.132 | <0.001 | 0.419 | 0.936 | 0.159 |
| NDV Child | Internalizing | 1.411 | 0.282 | 4.999 | <0.001 | 0.858 | 1.964 | 0.149 |
| NDV Child | Externalizing | 1.285 | 0.184 | 6.988 | <0.001 | 0.925 | 1.646 | 0.178 |

**Table S4b.** **Latent Behavioral Outcomes Regressed on Child NDV PGS, Including Paternal Age as a Covariate**

| **PGS** | **Domain** | **Estimate** | **SE** | **z** | **p** | **CI Lower** | **CI Upper** | **Std.Est** |
| --- | --- | --- | --- | --- | --- | --- | --- | --- |
| NDV Child | Social | 0.252 | 0.095 | 2.653 | 0.008 | 0.066 | 0.438 | 0.123 |
| NDV Child | Attention | 0.697 | 0.129 | 5.420 | <0.001 | 0.445 | 0.949 | 0.160 |
| NDV Child | Internalizing | 1.409 | 0.282 | 4.991 | <0.001 | 0.856 | 1.963 | 0.148 |
| NDV Child | Externalizing | 1.294 | 0.184 | 7.053 | <0.001 | 0.935 | 1.654 | 0.181 |

**Table S5a.** **Latent Behavioral Outcomes Regressed on Child Disorder-Specific PGSs**

| **PGS** | **Domain** | **Estimate** | **SE** | **z** | **p** | **CI Lower** | **CI Upper** | **Std.Est** |
| --- | --- | --- | --- | --- | --- | --- | --- | --- |
| ADHD Child | Social | 0.263 | 0.065 | 4.035 | <0.001 | 0.135 | 0.390 | 0.143 |
| ADHD Child | Attention | 0.552 | 0.097 | 5.690 | <0.001 | 0.362 | 0.743 | 0.188 |
| ADHD Child | Internalizing | 0.899 | 0.210 | 4.279 | <0.001 | 0.487 | 1.310 | 0.121 |
| ADHD Child | Externalizing | 1.046 | 0.142 | 7.353 | <0.001 | 0.768 | 1.325 | 0.198 |
| ASD Child | Social | 0.117 | 0.048 | 2.409 | 0.016 | 0.022 | 0.212 | 0.073 |
| ASD Child | Attention | 0.067 | 0.068 | 0.992 | 0.321 | -0.066 | 0.201 | 0.026 |
| ASD Child | Internalizing | 0.436 | 0.145 | 3.007 | 0.003 | 0.152 | 0.720 | 0.081 |
| ASD Child | Externalizing | 0.383 | 0.125 | 3.051 | 0.002 | 0.137 | 0.628 | 0.074 |
| MDD Child | Social | 0.162 | 0.051 | 3.154 | 0.002 | 0.061 | 0.263 | 0.102 |
| MDD Child | Attention | 0.315 | 0.082 | 3.825 | <0.001 | 0.153 | 0.476 | 0.105 |
| MDD Child | Internalizing | 0.907 | 0.167 | 5.441 | <0.001 | 0.580 | 1.234 | 0.157 |
| MDD Child | Externalizing | 0.531 | 0.116 | 4.593 | <0.001 | 0.305 | 0.758 | 0.124 |

**Table S5b.** **Latent Behavioral Outcomes Regressed on Child Disorder-Specific PGSs, Including Paternal Age as a Covariate**

| **PGS** | **Domain** | **Estimate** | **SE** | **z** | **p** | **CI Lower** | **CI Upper** | **Std.Est** |
| --- | --- | --- | --- | --- | --- | --- | --- | --- |
| ADHD Child | Social | 0.265 | 0.065 | 4.087 | <0.001 | 0.138 | 0.392 | 0.144 |
| ADHD Child | Attention | 0.566 | 0.095 | 5.938 | <0.001 | 0.379 | 0.753 | 0.189 |
| ADHD Child | Internalizing | 0.897 | 0.21 | 4.266 | <0.001 | 0.485 | 1.309 | 0.120 |
| ADHD Child | Externalizing | 1.052 | 0.142 | 7.391 | <0.001 | 0.773 | 1.331 | 0.200 |
| ASD Child | Social | 0.119 | 0.048 | 2.458 | 0.014 | 0.024 | 0.214 | 0.073 |
| ASD Child | Attention | 0.058 | 0.104 | 0.56 | 0.576 | -0.146 | 0.263 | 0.017 |
| ASD Child | Internalizing | 0.435 | 0.145 | 2.996 | 0.003 | 0.150 | 0.720 | 0.080 |
| ASD Child | Externalizing | 0.392 | 0.123 | 3.180 | 0.001 | 0.150 | 0.633 | 0.078 |
| MDD Child | Social | 0.165 | 0.052 | 3.189 | 0.001 | 0.064 | 0.266 | 0.102 |
| MDD Child | Attention | 0.325 | 0.08 | 4.036 | <0.001 | 0.167 | 0.482 | 0.103 |
| MDD Child | Internalizing | 0.906 | 0.167 | 5.440 | <0.001 | 0.579 | 1.232 | 0.157 |
| MDD Child | Externalizing | 0.540 | 0.115 | 4.677 | <0.001 | 0.313 | 0.766 | 0.126 |

**Table S6a.** **Brain Metrics Regressed on Child PGSs**

| **Outcome** | **PGS** | **Estimate** | **SE** | **p** | **Std.Est** |
| --- | --- | --- | --- | --- | --- |
| Cortical Surface Area | NDV Child | -0.056 | 0.025 | 0.026 | -0.031 |
| Cortical Surface Area | ADHD Child | -0.036 | 0.017 | 0.035 | -0.028 |
| Cortical Surface Area | ASD Child | -0.004 | 0.016 | 0.819 | -0.003 |
| Cortical Surface Area | MDD Child | 0.001 | 0.016 | 0.974 | 0.000 |
| Subcortical Gray Matter Volume | NDV Child | -0.005 | 0.030 | 0.875 | -0.003 |
| Subcortical Gray Matter Volume | ADHD Child | -0.007 | 0.021 | 0.739 | -0.006 |
| Subcortical Gray Matter Volume | ASD Child | 0.007 | 0.019 | 0.695 | 0.007 |
| Subcortical Gray Matter Volume | MDD Child | -0.056 | 0.010 | 0.002 | -0.051 |
| Cerebellum Volume | NDV Child | -0.039 | 0.039 | 0.308 | -0.022 |
| Cerebellum Volume | ADHD Child | -0.059 | 0.029 | 0.040 | -0.046 |
| Cerebellum Volume | ASD Child | 0.013 | 0.025 | 0.602 | 0.012 |
| Cerebellum Volume | MDD Child | -0.012 | 0.025 | 0.630 | -0.011 |

**Table S6b.** **Brain Metrics Regressed on Child PGSs, Including Paternal Age as a Covariate**

| **Outcome** | **PGS** | **Estimate** | **SE** | **p** | **Std.Est** |
| --- | --- | --- | --- | --- | --- |
| Cortical Surface Area | NDV Child | -0.056 | 0.025 | 0.026 | -0.031 |
| Cortical Surface Area | ADHD Child | -0.036 | 0.017 | 0.035 | -0.028 |
| Cortical Surface Area | ASD Child | -0.003 | 0.016 | 0.831 | -0.003 |
| Cortical Surface Area | MDD Child | 0.001 | 0.016 | 0.965 | 0.001 |
| Subcortical Gray Matter Volume | NDV Child | -0.005 | 0.030 | 0.880 | -0.003 |
| Subcortical Gray Matter Volume | ADHD Child | -0.007 | 0.021 | 0.735 | -0.006 |
| Subcortical Gray Matter Volume | ASD Child | 0.008 | 0.019 | 0.677 | 0.007 |
| Subcortical Gray Matter Volume | MDD Child | -0.056 | 0.019 | 0.003 | -0.051 |
| Cerebellum Volume | NDV Child | -0.039 | 0.039 | 0.313 | -0.022 |
| Cerebellum Volume | ADHD Child | -0.059 | 0.029 | 0.039 | -0.046 |
| Cerebellum Volume | ASD Child | 0.014 | 0.025 | 0.573 | 0.013 |
| Cerebellum Volume | MDD Child | -0.011 | 0.025 | 0.653 | -0.010 |

**Table S7a.** **Trio Models of NDV PGS Effects on Latent Behavioral Outcomes**

| **Domain** | **Trio member** | **PGS** | **Estimate** | **SE** | **z** | **p** | **Std.Est** |
| --- | --- | --- | --- | --- | --- | --- | --- |
| Social | child | NDV | 0.237 | 0.105 | 2.256 | 0.024 | 0.116 |
| Social | father | NDV | -0.015 | 0.076 | -0.200 | 0.842 | -0.006 |
| Social | mother | NDV | 0.044 | 0.076 | 0.583 | 0.560 | 0.019 |
| Attention | child | NDV | 0.543 | 0.181 | 3.005 | 0.003 | 0.128 |
| Attention | father | NDV | 0.115 | 0.155 | 0.740 | 0.459 | 0.024 |
| Attention | mother | NDV | 0.172 | 0.152 | 1.134 | 0.257 | 0.036 |
| Internalizing | child | NDV | 0.936 | 0.369 | 2.534 | 0.011 | 0.100 |
| Internalizing | father | NDV | 0.584 | 0.347 | 1.684 | 0.092 | 0.054 |
| Internalizing | mother | NDV | 0.472 | 0.348 | 1.358 | 0.174 | 0.045 |
| Externalizing | child | NDV | 0.986 | 0.271 | 3.644 | <0.001 | 0.140 |
| Externalizing | father | NDV | 0.243 | 0.256 | 0.950 | 0.342 | 0.030 |
| Externalizing | mother | NDV | 0.398 | 0.274 | 1.452 | 0.146 | 0.050 |

**Table S7b.** **Trio Models of NDV PGS Effects on Latent Behavioral Outcomes, Including Paternal Age as a Covariate**

| **Domain** | **Trio member** | **PGS** | **Estimate** | **SE** | **z** | **p** | **Std.Est** |
| --- | --- | --- | --- | --- | --- | --- | --- |
| Social | child | NDV | 0.240 | 0.106 | 2.267 | 0.023 | 0.117 |
| Social | mother | NDV | 0.045 | 0.077 | 0.579 | 0.562 | 0.019 |
| Social | father | NDV | -0.015 | 0.077 | -0.195 | 0.846 | -0.006 |
| Attention | child | NDV | 0.564 | 0.180 | 3.138 | 0.002 | 0.130 |
| Attention | mother | NDV | 0.174 | 0.155 | 1.119 | 0.263 | 0.036 |
| Attention | father | NDV | 0.113 | 0.160 | 0.704 | 0.482 | 0.023 |
| Internalizing | child | NDV | 0.934 | 0.369 | 2.530 | 0.011 | 0.099 |
| Internalizing | mother | NDV | 0.474 | 0.347 | 1.365 | 0.172 | 0.045 |
| Internalizing | father | NDV | 0.582 | 0.347 | 1.676 | 0.094 | 0.054 |
| Externalizing | child | NDV | 0.995 | 0.270 | 3.690 | <0.001 | 0.141 |
| Externalizing | mother | NDV | 0.390 | 0.274 | 1.425 | 0.154 | 0.050 |
| Externalizing | father | NDV | 0.255 | 0.256 | 0.996 | 0.319 | 0.032 |

**Table S8a.** **Trio Models of Disorder-Specific PGS Effects on Latent Behavioral Outcomes**

| **PGS** | **Domain** | **Trio Member** | **Estimate** | **SE** | **p** | **Z** | **Std.Est** |
| --- | --- | --- | --- | --- | --- | --- | --- |
| ADHD | Social | Child | 0.240 | 0.080 | 0.003 | 3.000 | 0.132 |
| ADHD | Social | Mother | -0.006 | 0.067 | 0.928 | -0.090 | -0.003 |
| ADHD | Social | Father | 0.052 | 0.066 | 0.427 | 0.788 | 0.028 |
| ADHD | Attention | Child | 0.551 | 0.124 | <0.001 | 4.444 | 0.188 |
| ADHD | Attention | Mother | -0.016 | 0.097 | 0.866 | -0.165 | -0.005 |
| ADHD | Attention | Father | 0.013 | 0.098 | 0.891 | 0.133 | 0.004 |
| ADHD | Internalizing | Child | 0.659 | 0.279 | 0.018 | 2.362 | 0.088 |
| ADHD | Internalizing | Mother | 0.231 | 0.254 | 0.362 | 0.909 | 0.030 |
| ADHD | Internalizing | Father | 0.268 | 0.250 | 0.285 | 1.072 | 0.035 |
| ADHD | Externalizing | Child | 0.878 | 0.195 | <0.001 | 4.503 | 0.168 |
| ADHD | Externalizing | Mother | 0.183 | 0.174 | 0.292 | 1.052 | 0.034 |
| ADHD | Externalizing | Father | 0.153 | 0.171 | 0.371 | 0.895 | 0.028 |
| ASD | Social | Child | 0.149 | 0.078 | 0.057 | 1.910 | 0.097 |
| ASD | Social | Mother | -0.007 | 0.060 | 0.910 | -0.117 | -0.004 |
| ASD | Social | Father | -0.067 | 0.057 | 0.241 | -1.175 | -0.043 |
| ASD | Attention | Child | 0.138 | 0.136 | 0.309 | 1.015 | 0.026 |
| ASD | Attention | Mother | -0.059 | 0.098 | 0.544 | -0.602 | -0.011 |
| ASD | Attention | Father | -0.192 | 0.097 | 0.047 | -1.979 | -0.034 |
| ASD | Internalizing | Child | 0.402 | 0.195 | 0.039 | 2.062 | 0.074 |
| ASD | Internalizing | Mother | 0.174 | 0.186 | 0.349 | 0.935 | 0.031 |
| ASD | Internalizing | Father | -0.094 | 0.176 | 0.593 | -0.534 | -0.017 |
| ASD | Externalizing | Child | 0.421 | 0.180 | 0.019 | 2.339 | 0.077 |
| ASD | Externalizing | Mother | -0.165 | 0.173 | 0.340 | -0.954 | -0.030 |
| ASD | Externalizing | Father | 0.065 | 0.162 | 0.687 | 0.401 | 0.012 |
| MDD | Social | Child | 0.065 | 0.060 | 0.279 | 1.083 | 0.043 |
| MDD | Social | Mother | 0.149 | 0.060 | 0.012 | 2.483 | 0.099 |
| MDD | Social | Father | 0.029 | 0.056 | 0.609 | 0.518 | 0.019 |
| MDD | Attention | Child | 0.148 | 0.111 | 0.183 | 1.333 | 0.050 |
| MDD | Attention | Mother | 0.291 | 0.097 | 0.003 | 3.000 | 0.098 |
| MDD | Attention | Father | 0.042 | 0.097 | 0.668 | 0.433 | 0.014 |
| MDD | Internalizing | Child | 0.506 | 0.216 | 0.019 | 2.343 | 0.093 |
| MDD | Internalizing | Mother | 0.547 | 0.182 | 0.003 | 3.005 | 0.099 |
| MDD | Internalizing | Father | 0.183 | 0.195 | 0.349 | 0.938 | 0.033 |
| MDD | Externalizing | Child | 0.286 | 0.152 | 0.059 | 1.882 | 0.074 |
| MDD | Externalizing | Mother | 0.379 | 0.134 | 0.005 | 2.828 | 0.098 |
| MDD | Externalizing | Father | 0.046 | 0.152 | 0.764 | 0.303 | 0.012 |

**Table S8b.** **Trio Models of Disorder-Specific PGS Effects on Latent Behavioral Outcomes, Including Paternal Age as a Covariate**

| **PGS** | **Domain** | **Trio Member** | **Estimate** | **SE** | **p** | **z** | **Std.Est** |
| --- | --- | --- | --- | --- | --- | --- | --- |
| ADHD | Social | Child | 0.242 | 0.081 | 0.003 | 3.005 | 0.132 |
| ADHD | Social | Mother | -0.007 | 0.067 | 0.918 | -0.103 | -0.004 |
| ADHD | Social | Father | 0.052 | 0.066 | 0.428 | 0.792 | 0.028 |
| ADHD | Attention | Child | 0.567 | 0.124 | <0.001 | 4.586 | 0.190 |
| ADHD | Attention | Mother | -0.017 | 0.099 | 0.866 | -0.169 | -0.005 |
| ADHD | Attention | Father | 0.010 | 0.101 | 0.920 | 0.101 | 0.003 |
| ADHD | Internalizing | Child | 0.656 | 0.280 | 0.019 | 2.344 | 0.087 |
| ADHD | Internalizing | Mother | 0.234 | 0.254 | 0.357 | 0.920 | 0.030 |
| ADHD | Internalizing | Father | 0.270 | 0.250 | 0.282 | 1.076 | 0.035 |
| ADHD | Externalizing | Child | 0.887 | 0.195 | <0.001 | 4.548 | 0.170 |
| ADHD | Externalizing | Mother | 0.175 | 0.174 | 0.315 | 1.005 | 0.032 |
| ADHD | Externalizing | Father | 0.153 | 0.171 | 0.370 | 0.897 | 0.028 |
| ASD | Social | Child | 0.153 | 0.078 | 0.050 | 1.964 | 0.098 |
| ASD | Social | Mother | -0.009 | 0.062 | 0.888 | -0.141 | -0.006 |
| ASD | Social | Father | -0.068 | 0.058 | 0.238 | -1.181 | -0.043 |
| ASD | Attention | Child | 0.146 | 0.115 | 0.207 | 1.263 | 0.027 |
| ASD | Attention | Mother | -0.065 | 0.093 | 0.487 | -0.696 | -0.012 |
| ASD | Attention | Father | -0.191 | 0.098 | 0.050 | -1.961 | -0.035 |
| ASD | Internalizing | Child | 0.400 | 0.196 | 0.042 | 2.038 | 0.073 |
| ASD | Internalizing | Mother | 0.177 | 0.186 | 0.340 | 0.954 | 0.032 |
| ASD | Internalizing | Father | -0.095 | 0.177 | 0.590 | -0.538 | -0.017 |
| ASD | Externalizing | Child | 0.435 | 0.177 | 0.014 | 2.463 | 0.082 |
| ASD | Externalizing | Mother | -0.164 | 0.172 | 0.339 | -0.956 | -0.031 |
| ASD | Externalizing | Father | 0.062 | 0.160 | 0.697 | 0.390 | 0.011 |
| MDD | Social | Child | 0.065 | 0.060 | 0.280 | 1.081 | 0.043 |
| MDD | Social | Mother | 0.152 | 0.060 | 0.012 | 2.512 | 0.100 |
| MDD | Social | Father | 0.030 | 0.057 | 0.596 | 0.531 | 0.019 |
| MDD | Attention | Child | 0.153 | 0.112 | 0.173 | 1.362 | 0.050 |
| MDD | Attention | Mother | 0.297 | 0.098 | 0.002 | 3.037 | 0.097 |
| MDD | Attention | Father | 0.046 | 0.098 | 0.639 | 0.469 | 0.015 |
| MDD | Internalizing | Child | 0.506 | 0.216 | 0.019 | 2.348 | 0.093 |
| MDD | Internalizing | Mother | 0.546 | 0.182 | 0.003 | 3.003 | 0.099 |
| MDD | Internalizing | Father | 0.182 | 0.195 | 0.352 | 0.931 | 0.032 |
| MDD | Externalizing | Child | 0.290 | 0.152 | 0.056 | 1.914 | 0.075 |
| MDD | Externalizing | Mother | 0.386 | 0.137 | 0.005 | 2.823 | 0.098 |
| MDD | Externalizing | Father | 0.058 | 0.152 | 0.702 | 0.383 | 0.015 |

**Table S9a.** **Informant-Specific Trio Models of MDD PGS Effects on Behavioral Outcomes**

| **PGS** | **Trio member** | **Domain** | **Rater** | **Estimate** | **SE** | **p** | **z** | **Est. Std.** |
| --- | --- | --- | --- | --- | --- | --- | --- | --- |
| MDD | child | Social | YSR | 0.054 | 0.083 | 0.517 | 0.648 | 0.021 |
| MDD | mother | Social | YSR | 0.120 | 0.078 | 0.126 | 1.531 | 0.046 |
| MDD | father | Social | YSR | 0.062 | 0.074 | 0.396 | 0.849 | 0.024 |
| MDD | child | Social | CBCL | 0.060 | 0.062 | 0.332 | 0.970 | 0.029 |
| MDD | mother | Social | CBCL | 0.153 | 0.059 | 0.010 | 2.573 | 0.074 |
| MDD | father | Social | CBCL | 0.014 | 0.055 | 0.792 | 0.264 | 0.007 |
| MDD | child | Attention | YSR | 0.163 | 0.116 | 0.160 | 1.405 | 0.047 |
| MDD | mother | Attention | YSR | 0.269 | 0.103 | 0.009 | 2.604 | 0.076 |
| MDD | father | Attention | YSR | 0.049 | 0.100 | 0.627 | 0.486 | 0.014 |
| MDD | child | Attention | CBCL | 0.093 | 0.106 | 0.384 | 0.871 | 0.027 |
| MDD | mother | Attention | CBCL | 0.238 | 0.097 | 0.014 | 2.448 | 0.069 |
| MDD | father | Attention | CBCL | 0.035 | 0.100 | 0.727 | 0.350 | 0.010 |
| MDD | child | Internalizing | YSR | 0.513 | 0.251 | 0.041 | 2.041 | 0.067 |
| MDD | mother | Internalizing | YSR | 0.391 | 0.224 | 0.081 | 1.746 | 0.051 |
| MDD | father | Internalizing | YSR | 0.358 | 0.206 | 0.083 | 1.735 | 0.045 |
| MDD | child | Internalizing | CBCL | 0.398 | 0.198 | 0.045 | 2.007 | 0.063 |
| MDD | mother | Internalizing | CBCL | 0.585 | 0.183 | 0.001 | 3.190 | 0.092 |
| MDD | father | Internalizing | CBCL | 0.049 | 0.176 | 0.780 | 0.280 | 0.008 |
| MDD | child | Externalizing | YSR | 0.225 | 0.180 | 0.211 | 1.250 | 0.040 |
| MDD | mother | Externalizing | YSR | 0.231 | 0.160 | 0.150 | 1.440 | 0.041 |
| MDD | father | Externalizing | YSR | 0.215 | 0.158 | 0.172 | 1.365 | 0.037 |
| MDD | child | Externalizing | CBCL | 0.330 | 0.174 | 0.058 | 1.894 | 0.061 |
| MDD | mother | Externalizing | CBCL | 0.489 | 0.174 | 0.005 | 2.815 | 0.089 |
| MDD | father | Externalizing | CBCL | -0.049 | 0.156 | 0.753 | -0.315 | -0.009 |

**Table S9b.** **Informant-Specific Trio Models of MDD PGS Effects on Behavioral Outcomes, Including Paternal Age as a Covariate**

| **PGS** | **Trio member** | **Domain** | **Rater** | **Estimate** | **SE** | **p** | **z** | **Est. Std.** |
| --- | --- | --- | --- | --- | --- | --- | --- | --- |
| MDD | child | Social | YSR | 0.055 | 0.083 | 0.513 | 0.654 | 0.021 |
| MDD | mother | Social | YSR | 0.121 | 0.078 | 0.123 | 1.542 | 0.046 |
| MDD | father | Social | YSR | 0.065 | 0.074 | 0.377 | 0.883 | 0.025 |
| MDD | child | Social | CBCL | 0.060 | 0.062 | 0.331 | 0.972 | 0.030 |
| MDD | mother | Social | CBCL | 0.153 | 0.059 | 0.010 | 2.571 | 0.074 |
| MDD | father | Social | CBCL | 0.015 | 0.055 | 0.787 | 0.270 | 0.007 |
| MDD | child | Attention | YSR | 0.165 | 0.116 | 0.156 | 1.417 | 0.047 |
| MDD | mother | Attention | YSR | 0.271 | 0.103 | 0.009 | 2.626 | 0.076 |
| MDD | father | Attention | YSR | 0.056 | 0.100 | 0.578 | 0.556 | 0.015 |
| MDD | child | Attention | CBCL | 0.093 | 0.106 | 0.382 | 0.874 | 0.027 |
| MDD | mother | Attention | CBCL | 0.239 | 0.097 | 0.014 | 2.458 | 0.070 |
| MDD | father | Attention | CBCL | 0.036 | 0.100 | 0.716 | 0.364 | 0.010 |
| MDD | child | Internalizing | YSR | 0.513 | 0.251 | 0.041 | 2.040 | 0.067 |
| MDD | mother | Internalizing | YSR | 0.390 | 0.224 | 0.082 | 1.740 | 0.051 |
| MDD | father | Internalizing | YSR | 0.355 | 0.206 | 0.085 | 1.722 | 0.045 |
| MDD | child | Internalizing | CBCL | 0.398 | 0.198 | 0.045 | 2.007 | 0.063 |
| MDD | mother | Internalizing | CBCL | 0.585 | 0.184 | 0.001 | 3.182 | 0.091 |
| MDD | father | Internalizing | CBCL | 0.048 | 0.176 | 0.785 | 0.273 | 0.007 |
| MDD | child | Externalizing | YSR | 0.227 | 0.180 | 0.206 | 1.265 | 0.040 |
| MDD | mother | Externalizing | YSR | 0.234 | 0.160 | 0.144 | 1.461 | 0.041 |
| MDD | father | Externalizing | YSR | 0.226 | 0.158 | 0.153 | 1.430 | 0.039 |
| MDD | child | Externalizing | CBCL | 0.331 | 0.174 | 0.057 | 1.903 | 0.061 |
| MDD | mother | Externalizing | CBCL | 0.495 | 0.174 | 0.004 | 2.841 | 0.090 |
| MDD | father | Externalizing | CBCL | -0.042 | 0.157 | 0.790 | -0.267 | -0.007 |

**Table S10a.** **NDV Trio Mediation Effects on Latent Behavioral Outcomes**

| **Pathway** | **Domain** | **Brain metric** | **Estimate** | **SE** | **z** | **p** | **Std. Est.** |
| --- | --- | --- | --- | --- | --- | --- | --- |
| Father, Indirect | Social | Cortical surface area | 0.000 | 0.006 | -0.046 | 0.964 | 0.000 |
| Mother, Indirect | Social | Cortical surface area | -0.007 | 0.024 | -0.270 | 0.787 | -0.007 |
| Child, Indirect | Social | Cortical surface area | 0.012 | 0.044 | 0.283 | 0.777 | 0.014 |
| Father, Indirect | Attention | Cortical surface area | -0.002 | 0.010 | -0.195 | 0.846 | -0.001 |
| Mother, Indirect | Attention | Cortical surface area | -0.012 | 0.012 | -1.069 | 0.285 | -0.008 |
| Child, Indirect | Attention | Cortical surface area | 0.023 | 0.015 | 1.541 | 0.123 | 0.018 |
| Father, Indirect | Internalizing | Cortical surface area | 0.000 | 0.004 | -0.117 | 0.907 | 0.000 |
| Mother, Indirect | Internalizing | Cortical surface area | 0.004 | 0.008 | 0.490 | 0.624 | 0.003 |
| Child, Indirect | Internalizing | Cortical surface area | -0.008 | 0.015 | -0.524 | 0.600 | -0.006 |
| Father, Indirect | Externalizing | Cortical surface area | 0.000 | 0.007 | 0.001 | 0.999 | 0.000 |
| Mother, Indirect | Externalizing | Cortical surface area | -0.008 | 0.011 | -0.719 | 0.472 | -0.005 |
| Child, Indirect | Externalizing | Cortical surface area | 0.016 | 0.019 | 0.834 | 0.404 | 0.011 |
| Father, Indirect | Social | Subcortical GMV | 0.000 | 0.001 | 0.117 | 0.907 | 0.000 |
| Mother, Indirect | Social | Subcortical GMV | 0.001 | 0.003 | 0.263 | 0.792 | 0.001 |
| Child, Indirect | Social | Subcortical GMV | -0.002 | 0.007 | -0.273 | 0.785 | -0.002 |
| Father, Indirect | Attention | Subcortical GMV | 0.000 | 0.001 | 0.004 | 0.997 | 0.000 |
| Mother, Indirect | Attention | Subcortical GMV | 0.000 | 0.003 | 0.004 | 0.997 | 0.000 |
| Child, Indirect | Attention | Subcortical GMV | 0.000 | 0.006 | -0.004 | 0.997 | 0.000 |
| Father, Indirect | Internalizing | Subcortical GMV | 0.000 | 0.003 | 0.091 | 0.928 | 0.000 |
| Mother, Indirect | Internalizing | Subcortical GMV | 0.002 | 0.004 | 0.497 | 0.619 | 0.001 |
| Child, Indirect | Internalizing | Subcortical GMV | -0.004 | 0.006 | -0.631 | 0.528 | -0.003 |
| Father, Indirect | Externalizing | Subcortical GMV | 0.000 | 0.001 | 0.071 | 0.944 | 0.000 |
| Mother, Indirect | Externalizing | Subcortical GMV | 0.000 | 0.003 | 0.077 | 0.938 | 0.000 |
| Child, Indirect | Externalizing | Subcortical GMV | 0.000 | 0.006 | -0.077 | 0.938 | 0.000 |
| Father, Indirect | Social | Cerebellum Cortical Volume | -0.001 | 0.003 | -0.264 | 0.792 | -0.001 |
| Mother, Indirect | Social | Cerebellum Cortical Volume | -0.001 | 0.004 | -0.267 | 0.790 | -0.001 |
| Child, Indirect | Social | Cerebellum Cortical Volume | 0.001 | 0.005 | 0.268 | 0.789 | 0.002 |
| Father, Indirect | Attention | Cerebellum Cortical Volume | -0.007 | 0.006 | -1.152 | 0.249 | -0.005 |
| Mother, Indirect | Attention | Cerebellum Cortical Volume | -0.010 | 0.007 | -1.345 | 0.179 | -0.007 |
| Child, Indirect | Attention | Cerebellum Cortical Volume | 0.013 | 0.009 | 1.467 | 0.142 | 0.010 |
| Father, Indirect | Internalizing | Cerebellum Cortical Volume | -0.006 | 0.006 | -0.951 | 0.342 | -0.004 |
| Mother, Indirect | Internalizing | Cerebellum Cortical Volume | -0.008 | 0.007 | -1.094 | 0.274 | -0.005 |
| Child, Indirect | Internalizing | Cerebellum Cortical Volume | 0.010 | 0.009 | 1.147 | 0.251 | 0.008 |
| Father, Indirect | Externalizing | Cerebellum Cortical Volume | -0.007 | 0.006 | -1.114 | 0.265 | -0.005 |
| Mother, Indirect | Externalizing | Cerebellum Cortical Volume | -0.010 | 0.007 | -1.332 | 0.183 | -0.007 |
| Child, Indirect | Externalizing | Cerebellum Cortical Volume | 0.013 | 0.009 | 1.397 | 0.162 | 0.010 |

**Table S10b.** **NDV Trio Mediation Effects on Latent Behavioral Outcomes, Including Paternal Age as a Covariate**

| **Pathway** | **Domain** | **Brain metric** | **Estimate** | **SE** | **Z** | **p** | **Std. Est.** |
| --- | --- | --- | --- | --- | --- | --- | --- |
| Father, Indirect | Social | Cortical surface area | 0.000 | 0.006 | -0.046 | 0.963 | 0.000 |
| Mother, Indirect | Social | Cortical surface area | -0.007 | 0.024 | -0.281 | 0.779 | -0.007 |
| Child, Indirect | Social | Cortical surface area | 0.013 | 0.043 | 0.295 | 0.768 | 0.015 |
| Father, Indirect | Attention | Cortical surface area | -0.002 | 0.010 | -0.189 | 0.850 | -0.001 |
| Mother, Indirect | Attention | Cortical surface area | -0.012 | 0.012 | -1.063 | 0.288 | -0.008 |
| Child, Indirect | Attention | Cortical surface area | 0.023 | 0.015 | 1.532 | 0.125 | 0.017 |
| Father, Indirect | Internalizing | Cortical surface area | 0.000 | 0.004 | -0.117 | 0.907 | 0.000 |
| Mother, Indirect | Internalizing | Cortical surface area | 0.004 | 0.008 | 0.487 | 0.626 | 0.003 |
| Child, Indirect | Internalizing | Cortical surface area | -0.008 | 0.015 | -0.521 | 0.603 | -0.006 |
| Father, Indirect | Externalizing | Cortical surface area | 0.000 | 0.007 | 0.004 | 0.997 | 0.000 |
| Mother, Indirect | Externalizing | Cortical surface area | -0.008 | 0.011 | -0.720 | 0.471 | -0.005 |
| Child, Indirect | Externalizing | Cortical surface area | 0.016 | 0.019 | 0.836 | 0.403 | 0.012 |
| Father, Indirect | Social | Subcortical GMV | 0.000 | 0.001 | 0.116 | 0.907 | 0.000 |
| Mother, Indirect | Social | Subcortical GMV | 0.001 | 0.003 | 0.273 | 0.785 | 0.001 |
| Child, Indirect | Social | Subcortical GMV | -0.002 | 0.007 | -0.285 | 0.775 | -0.002 |
| Father, Indirect | Attention | Subcortical GMV | 0.000 | 0.001 | -0.025 | 0.980 | 0.000 |
| Mother, Indirect | Attention | Subcortical GMV | 0.000 | 0.003 | -0.026 | 0.979 | 0.000 |
| Child, Indirect | Attention | Subcortical GMV | 0.000 | 0.006 | 0.026 | 0.979 | 0.000 |
| Father, Indirect | Internalizing | Subcortical GMV | 0.000 | 0.002 | 0.091 | 0.927 | 0.000 |
| Mother, Indirect | Internalizing | Subcortical GMV | 0.002 | 0.004 | 0.496 | 0.620 | 0.001 |
| Child, Indirect | Internalizing | Subcortical GMV | -0.004 | 0.006 | -0.629 | 0.529 | -0.003 |
| Father, Indirect | Externalizing | Subcortical GMV | 0.000 | 0.001 | 0.081 | 0.936 | 0.000 |
| Mother, Indirect | Externalizing | Subcortical GMV | 0.000 | 0.003 | 0.092 | 0.926 | 0.000 |
| Child, Indirect | Externalizing | Subcortical GMV | -0.001 | 0.006 | -0.093 | 0.926 | 0.000 |
| Father, Indirect | Social | Cerebellum Cortical Volume | -0.001 | 0.003 | -0.270 | 0.787 | -0.001 |
| Mother, Indirect | Social | Cerebellum Cortical Volume | -0.001 | 0.004 | -0.273 | 0.785 | -0.001 |
| Child, Indirect | Social | Cerebellum Cortical Volume | 0.002 | 0.006 | 0.274 | 0.784 | 0.002 |
| Father, Indirect | Attention | Cerebellum Cortical Volume | -0.007 | 0.006 | -1.147 | 0.251 | -0.005 |
| Mother, Indirect | Attention | Cerebellum Cortical Volume | -0.010 | 0.008 | -1.336 | 0.181 | -0.007 |
| Child, Indirect | Attention | Cerebellum Cortical Volume | 0.013 | 0.009 | 1.457 | 0.145 | 0.010 |
| Father, Indirect | Internalizing | Cerebellum Cortical Volume | -0.006 | 0.006 | -0.944 | 0.345 | -0.004 |
| Mother, Indirect | Internalizing | Cerebellum Cortical Volume | -0.008 | 0.007 | -1.085 | 0.278 | -0.005 |
| Child, Indirect | Internalizing | Cerebellum Cortical Volume | 0.010 | 0.009 | 1.137 | 0.256 | 0.008 |
| Father, Indirect | Externalizing | Cerebellum Cortical Volume | -0.007 | 0.006 | -1.134 | 0.257 | -0.005 |
| Mother, Indirect | Externalizing | Cerebellum Cortical Volume | -0.010 | 0.008 | -1.363 | 0.173 | -0.007 |
| Child, Indirect | Externalizing | Cerebellum Cortical Volume | 0.013 | 0.009 | 1.434 | 0.151 | 0.010 |
